# Epigenetic Modifiers as Potential Therapeutic Targets in Diabetic Kidney Disease

**DOI:** 10.3390/ijms21114113

**Published:** 2020-06-09

**Authors:** Julio M. Martinez-Moreno, Miguel Fontecha-Barriuso, Diego Martin-Sanchez, Juan Guerrero-Mauvecin, Elena Goma-Garces, Beatriz Fernandez-Fernandez, Sol Carriazo, Maria D. Sanchez-Niño, Adrian M. Ramos, Marta Ruiz-Ortega, Alberto Ortiz, Ana B. Sanz

**Affiliations:** 1 IIS-Fundacion Jimenez Diaz, Av Reyes Católicos 2, 28040 Madrid, Spain; juliomanuelm@gmail.com (J.M.M.-M.); miguel.fontecha@quironsalud.es (M.F.-B.); diego.martin@fjd.es (D.M.-S.); juang.mauvecin@quironsalud.es (J.G.-M.); elena.goma@quironsalud.es (E.G.-G.); BFernandez@fjd.es (B.F.-F.); sol.carriazo@quironsalud.es (S.C.); mdsanchez@fjd.es (M.D.S.-N.); AMRamos@fjd.es (A.M.R.); MRuizO@quironsalud.es (M.R.-O.); 2 Red de Investigación Renal, 28029 Madrid, Spain; 3 School of Medicine, Arturo Michelena University, 28040 Madrid, Spain; 4 Instituto de Reina Sofia de Investigación Renal, 28040 Madrid, Spain

**Keywords:** diabetes, diabetic kidney disease, epigenetic, crotonylation, apabetalone, BET, DNA methylation, chronic kidney disease

## Abstract

Diabetic kidney disease is one of the fastest growing causes of death worldwide. Epigenetic regulators control gene expression and are potential therapeutic targets. There is functional interventional evidence for a role of DNA methylation and the histone post-translational modifications—histone methylation, acetylation and crotonylation—in the pathogenesis of kidney disease, including diabetic kidney disease. Readers of epigenetic marks, such as bromodomain and extra terminal (BET) proteins, are also therapeutic targets. Thus, the BD2 selective BET inhibitor apabetalone was the first epigenetic regulator to undergo phase-3 clinical trials in diabetic kidney disease with an endpoint of kidney function. The direct therapeutic modulation of epigenetic features is possible through pharmacological modulators of the specific enzymes involved and through the therapeutic use of the required substrates. Of further interest is the characterization of potential indirect effects of nephroprotective drugs on epigenetic regulation. Thus, SGLT2 inhibitors increase the circulating and tissue levels of β-hydroxybutyrate, a molecule that generates a specific histone modification, β-hydroxybutyrylation, which has been associated with the beneficial health effects of fasting. To what extent this impact on epigenetic regulation may underlie or contribute to the so-far unclear molecular mechanisms of cardio- and nephroprotection offered by SGLT2 inhibitors merits further in-depth studies.

## 1. Diabetic Kidney Disease Outcomes: An Unmet Medical Need

According to the Global Burden of Disease (GBD) study, the burden of non-communicable diseases is increasing and now accounts for 73% of global deaths [1]. Indeed, 50% of all global deaths (28.8 million), were attributable to four risk factors: high blood glucose, high blood pressure, smoking, and high body-mass index [2]. The prevalence of diabetes mellitus is also increasing and diabetes, by causing Diabetic Kidney Disease (DKD), is the leading driver of Chronic Kidney Disease (CKD) [1]. Patients with DKD-CKD or end stage renal disease (ESRD), have higher mortality rates than non-DKD patients [3]. CKD secondary to type 1 and type 2 diabetes resulted in 2.9 million (2.4–3.5) disability-adjusted life-years (DALYs) and 8.1 million (7.1–9.2) DALYs [1]. Worldwide deaths attributed to DKD were estimated at 219,451 in 2017 [1]. While mortality due to DKD is increasing, other CKD causes are remaining relatively stable, reflecting the increase in prevalence of DKD-CKD, which is outpacing other forms of CKD [1]. By 2040, CKD is expected to become the fifth most common cause of death worldwide, with DKD being the first global cause of CKD [4]. The burden of DKD is growing at a much faster rate in low- and middle-income countries than in high-income countries [1]. Improving the clinical management of diabetes and DKD could translate into the modification of current global mortality trends. In this regard, the optimal therapeutic approach to DKD is an unmet medical need.

**Figure 1:**
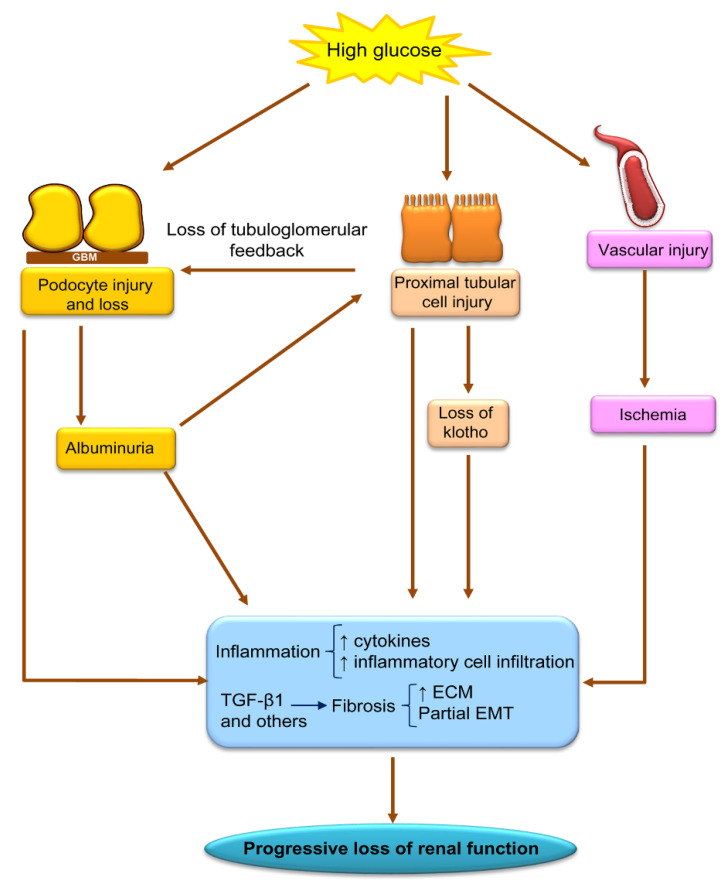
Key pathophysiological features of Diabetic Kidney Disease (DKD), emphasizing key processes and cell types as well as some of the multiple molecules involved. ECM: increase extracellular matrix. EMT: epithelial-to-mesenchymal transition.

DKD is heralded by increased glomerular filtration likely resulting from hyperglycemia-induced proximal tubular dysfunction and loss of the tubuloglomerular feedback [5]. This is followed by increasing albuminuria, progressing to overt proteinuria and loss of kidney function reaching end-stage kidney disease after around 10 years. Thus, DKD was classically considered a proteinuric form of CKD and, although recently, non-proteinuric DKD has been increasingly described, and proteinuric patients have worse outcomes [6]; this places podocyte injury and loss as a key contributor to DKD progression (Figure 1). High glucose concentrations and hemodynamic changes driven by proximal tubular cell glucose overload are key drivers of podocyte injury [7,8]. Hyperglycemia also damages vascular and tubular cells. On top of hyperglycemia-induced tubular cell injury, albuminuria itself also damages tubular cells, decreasing the expression of the nephroprotective and anti-aging protein Klotho, as well as eliciting proinflammatory and profibrotic responses [8,9,10,11,12]. Fibrosis is an early event, initially characterized by thickened epithelial basement membranes but eventually evolving to glomerulosclerosis and interstitial fibrosis. Fibrinogenic factors such as TGF-β1 and events such as incomplete epithelial-to-mesenchymal transition (EMT) contribute to increased extracellular matrix (ECM) production.

Recently, inhibitors of SGLT2i—oral antidiabetic drugs that improve glycemic control by decreasing renal glucose reabsorption in proximal tubules and increasing glycosuria—were shown to improve kidney outcomes and cardiovascular outcomes in patients with type 2 diabetes and/or heart failure [5,13]. In particular, canagliflozin improved kidney outcomes in patients with DKD [14]. As a consequence, leading clinical guidelines recommend their preferential use in diabetic patients with high cardiovascular or renal risk [5,15]. The current hypothesis is that their generalized use may improve the dismal outcome of DKD patients. However, there is still a residual risk of cardiovascular death and CKD progression [5].

We now review a recent and promising aspect of the pathogenesis of DKD: the role of epigenetic regulation in kidney injury. This field has already reached the clinic and obtained promising results in randomized clinical trials. In this regard, the study of epigenetic changes in DKD may identify new therapeutic targets by studying layers of gene expression regulation other than mRNA and translation.

## 2. Epigenetic Regulation of Gene Expression 

**Figure 2:**
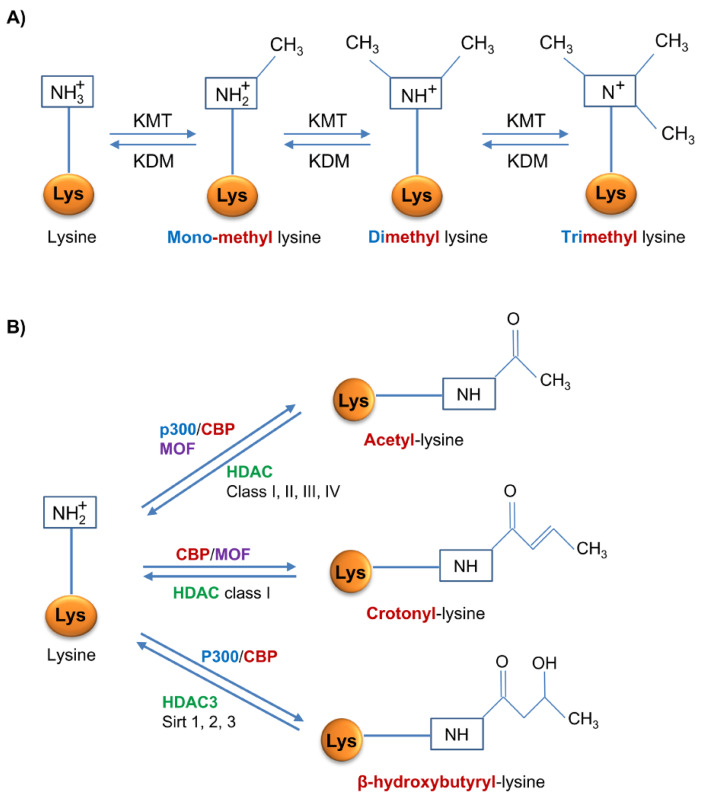
Enzymatic regulation of epigenetic histone modifications most relevant in diabetic nephropathy. (**A**) Lysine mono-, di- or tri- methylation is mediated by lysine methyl-transferases (KMT) and demethylation by lysine demethylases (KDM). (**B**) Histone acetylation, crotonylation and β-hydroxybutyrylation share some enzymes such as the histone acyl transferase CBP and combinations of p300 and MOF, and some histone deacetylases (HDACs) that may also remove other acyl groups. CBP: CREB-binding protein; MOF: Males absent on the first.

Epigenetics refers to heritable changes in gene expression patterns that are not caused by a specific DNA nucleotide sequence itself. Epigenetic information is both heritable/self-perpetuating and dynamic and reversible in response to a changing environment. The main epigenetic regulators are DNA methylation and histone post-translational modifications [16,17]. The best characterized histone post-translational modifications from a functional point of view relevant for kidney disease are lysine histone methylation, acetylation and crotonylation (Figure 2). On top of these, histone β-hydroxybutyrylation may be of interest when discussing DKD. Additionally, it should be acknowledged that over 100 histone modifications have been described, 67 of them in a single publication and new modifications keep being described, such as lactylation [18,19,20]. Some authors also include miRNAs, but these will not be discussed in the present review. Additionally, epigenetic readers that identify and interpret epigenetic signals, are key components of the system.

### 2.1. DNA Methylation

DNA methylation is an enzymatic process that involves the covalent transfer of a methyl group (CH_3_) from S-adenosyl L methionine (SAM) to the 5-carbon of cytosine residues on CpG sites [21], mainly on those located in CpG islands (800–1000 nucleotides) present in the gene promoter or in the first exons [22]. CpG methylation in gene promoters is associated with transcriptional inhibition due to both the recruitment of co-repressors and by impeding transcription factor binding by packaging chromatin [23]. However, DNA methylation in the gene body promotes gene expression through modulation of transcription elongation and RNA splicing [24].

Methylation profiles are tissue specific and define cell fate, which explains the different cell identities and their expression patterns despite their common genome. This process is catalyzed by DNA methyltransferases (DNMT). DNMT1 controls the maintenance of methylation marks acting on hemimethylated DNA during DNA replication or DNA repair in somatic cells, while DNMT3a and DNMT3b are involved in de novo methylation. DNMT3a is relatively ubiquitous and is associated with normal cell differentiation, while DNMT3b is generally absent in adult tissues and is necessary for early development [22]. In this sense, the methylation patterns during embryonic development are established by the DNMT3 subfamily and are further maintained through somatic divisions by DNMT1 in differentiated cells [25].

Epigenetic prints were firstly considered as relatively stable marks until the discovery of the Ten-eleven-translocation (TET) family proteins involved in DNA demethylation. These enzymes catalyze the oxidation of 5-methyl cytosine (5mC) to form 5-hydroxymethyl cytosine (5hmC) and other subsequent oxidized products. DNA repair enzymes exscind these oxidized products and incorporate unmethylated cytosines. DNA demethylation can also occur passively by preventing DNMT1 binding during DNA replication [16,26]. 

### 2.2. Histone Methylation

Methylation involves the covalent addition of methyl groups to lysine (Lys) and arginine (Arg) residues of histones. This process is regulated by a large number of coordinated enzymes that regulate gene expression, and genomic stability [27]. Histone methylation depends on histone methyltransferases (HMTs), grouped into lysine-specific (KMTs) and arginine-specific (PRMTs) methyltransferases. Lys may be mono-, di- or tri-methylated, whereas Arg may be symmetrically or asymmetrically mono or dimethylated [28]. KMTs are divided in two classes based on their catalytic domain structure, the SET domain containing enzymes, and the DOT1L/KMT4 family [29].

Histone methylation has different potential impacts on transcription. Arg methylation promotes transcriptional activation, while Lys methylation may activate or repress transcription depending on the methylation site [30]. For instance, monomethylation, dimethylation or trimethylation of H3 at Lys4 (H3K4m1/2/3) are associated to transcriptionally active genome regions, while trimethylation of H3 at Lys9 or at Lys27 (H3K9m3/H3K27m3) and trimethylation of H4 at Lys20 (H4K20m3) are enriched in repressed regions.

Histone methylation was considered stable until the report of Lys-specific demethylase 1 (KDM1A) in 2004 [31]. Thus, histone lysine demethylases (KDM) and histone arginine demethylases are enzymes that remove methyl modifications from Lys or Arg respectively.

### 2.3. Histone Acetylation

Histone acetyltransferases (HATs), such as CREB-binding protein (CBP) and p300, transfer an acetyl group (COCH_3_) from acetyl-coenzyme A (acetyl-CoA) to Lys residues in histones, neutralizing their positive charge and relaxing the chromatin [32]. Given the need for acetyl-CoA, histone acetylation may also be regulated metabolically by acetyl-CoA availability [32]. Histone acetylation mostly occurs in promoters and enhancer of target genes promoting their expression [33,34]. Four classes of histone deacetylases (HDAC) remove acetyl groups. Class I HDAC are ubiquitous nuclear enzymes that regulate cell survival and proliferation. Class II HDACs, localized in nuclei and cytosol, have tissue-specific roles. Class III HDACs are the sirtuins, that regulate numerous physiological and pathological processes. There is only one class IV HDAC: HDAC11 [35]. 

### 2.4. Histone Crotonylation

Histone lysine crotonylation (Kcr) is a recently described post-transcriptional modification of histones, which consists in the addition of a crotonyl group from crotonyl-CoA to Lys residues and is critically important for global transcriptional regulation in mammalian cells [20,36]. Kcr is evolutionarily conserved and marks either active promoters or potential enhancers, having a different genomic pattern than histone lysine acetylation (Kac), although the impact on gene expression is unclear, as it can activate or repress gene transcription [20,37]. Kcr shares enzyme regulators with Kac, but it is mechanistically and functionally different [20,38]. Thus, acetyltransferases such us CBP or the evolutionary conserved MOF have crotonyltransferase activity but CBP-catalyzed histone crotonylation directly stimulates transcription to a greater degree than histone acetylation [38,39]. HDACs, and more specifically class I HDACs, also have decrotonylase activity, although Kcr is more resistant to deacylation than Kac, supporting the idea of a robust transcription [40,41]. Kcr is also regulated by metabolic pathways that control crotonate availability [42]. Crotonate is the short-chain fatty acid precursor of crotonyl-CoA, a chemical process catalyzed by the Acyl-CoA Synthetase Short Chain Family Member 2 (ACSS2) [38]. In cultured kidney cells, crotonate availability was associated with increased or decreased gene expression of genes involved in the pathogenesis of kidney disease [42,43]. 

### 2.5. Histone β-Hydroxybutyrylation

β-hydroxybutyrate (BHB) is the most abundant ketone body, representing around 70–80% of the total, and is mostly produced in the liver via fatty acid metabolism in situations where glucose level is too low and the body needs energy, such as during dietary restriction, periods of fasting or prolonged intense exercise [44]. Moreover, BHB administration protects against oxidative stress and it exerts anti-inflammatory and anti-oxidative properties [45,46]. In cells, high levels of BHB favor lysine β-hydroxybutyrylation (Kbhb), a recently described post-translational modification of histones [47,48] and other proteins such as p53 [49]. In p53, Kbhb decreases p53 activity because it replaces an acetyl mark [49]. Few studies have explored the influence of BHB and (Kbhb) on several molecular processes in different cells and tissues. In livers from either mouse subjected to prolonged fasting or with streptozotocin-induced diabetic ketoacidosis, as well as in human cells cultured with BHB, histone Kbhb marks are related to active gene promoters of metabolic pathways induced by ketone acids. This is the first time that histone Kbhb was identified as a new way of epigenetic regulation that regulates cellular physiology [47]. Ketogenesis also represents a metabolic pathway required for the development of memory CD8+ T cells where BHB promotes β-hydroxybutyrylation of Lys9 in H3 (H3K9bhb) and this is associated to upregulation in the gene expression of *Foxo1* and *Ppargc1a*, which cooperatively upregulate *Pck1* expression [50]. Furthermore, BHB regulates the inflammasome and the expression of associated inflammatory genes through histone Kbhb, reducing inflammatory responses and blood pressure [51]. Finally, BHB induces β-hydroxybutyrylation in histone H3 (H3K9bhb) of the adiponectin gene and adiponectin expression in adipocytes [52]. As adiponectin also has anti-inflammatory and anti-atherogenic properties [53], BHB may be protective through modulation of epigenetic regulation [52].

β-hydroxybutyrylation of lysine residues is catalyzed by CBP and p300 [49,54]. On the other hand, SIRT3 and HDAC3 show strong activity in removing β-hydroxybutyryl groups from lysines in histones. However, SIRT3 is unable to remove β-hydroxybutyryl marks flanked by glycine, unlike HDAC3, which removes β-hydroxybutyryl marks regardless of adjacent glycines. SIRT1 and SIRT22 may also catalyze β-hydroxybutyryl group hydrolysis from lysines in histones [55,56].

### 2.6. Epigenetic Readers

“Readers” identify epigenetic modifications and influence gene expression in a highly specific manner for the residue and the degree of histone methylation (chromodomain and bromodomain proteins) or acetylation [27,57,58,59]. Bromodomains are a highly conserved motif of 110 amino acids with protein interaction functions. They are involved in chromatin remodeling and transcriptional regulation [58,59]. The bromodomain and extra terminal (BET) protein family (BRD2, BRD3, BRD4 and BRDT) are epigenetic readers that, via bromodomains (BD) 1 and 2, regulate gene transcription by binding to acetylated Lys residues.

## 3. DNA Methylation in Diabetic Kidney Disease

**Figure 3:**
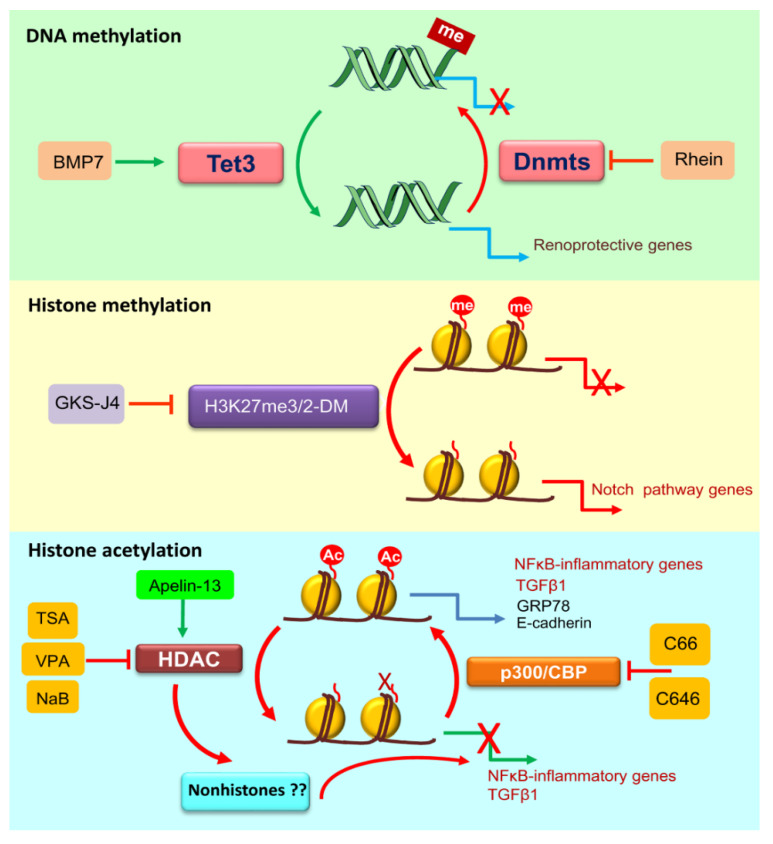
Summary of therapeutic intervention on epigenetic modifications with evidence of renal benefit in preclinical diabetic nephropathy. Different approaches targeting epigenetic modifications attenuate renal injury in experimental models DN. me, methylation; Ac, acetylation; TSA, trichostatin A; VPA, valproic acid; NaB, sodium butyrate. Inhibition of DNA methylases or activation of DNA demethylases, inhibition of specific histone demethylases, and inhibition of certain histone deacetylases (HDAC) (e.g., HDAC2 by TSA and VPA and NaB unknown), activation of other HDACs (e.g., HDAC1 by apelin-13) or activation of histone acetylases such as p300/CBP were all protective in preclinical DN.

Different methylated patterns in key regulatory genes are thought to contribute to the development of DKD and specific enzymes implicated in histones methylation might play a role in diabetic nephropathy (Figure 3) (Table 1) [60]. This is also the case for other forms of CKD. In tubules from human CKD kidneys (50% with DKD), most of the differentially methylated regions were located in enhancers and were correlated with the increased expression of key fibrotic genes [61]. In addition, methylation in CpG islands of different genes involved in pathways known to promote the epithelial-to-mesenchymal transition (EMT), were associated with rapid loss of kidney function in CKD patients (again 50% with DKD) [62]. 

Altered DNA methylation in intrinsic renal cells and in leukocytes may contribute to DKD progression. In proximal tubules from *db*/*db* mice, a mouse model of leptin deficiency widely used as model of type 2 diabetes (T2D), genes involved in glucose metabolism were aberrantly DNA methylated [63]. Similarly, cultured proximal tubular cells exposed to high glucose concentrations and kidneys from mice with type 1 streptozotocin-induced diabetes mice exhibited DNA hypomethylation of *MIOX*, which was associated with enhanced binding of the transcription factor SP1 at the promoter of genes involved in oxidative stress, hypoxia and fibrosis [64]. Aberrant DNA methylation patterns were also observed in cultured podocytes exposed to high glucose concentrations [65]. The *MMP9* promoter region in podocytes contained demethylated CpG sites, and high glucose reduced *MMP9* promoter methylation, thus increasing its promoter activity, and contributing to podocyte EMT [66]. Transcriptional repression of the transcription factor Kruppel-like factor 4 (KLF4) in podocytes was associated with increased DNA methylation at the nephrin (*Nphs1*) promoter, leading to podocyte apoptosis and proteinuria in *db*/*db* mice, whereas KLF4 overexpression has renoprotective effects [67]. KAT5-mediated DNA repair is essential for podocyte maintenance and is related to changes in DNA methylation status [68]. Interestingly, crosstalk between proximal tubules and podocytes may depend on epigenetic mechanisms. Tubule-specific overexpression of SIRT1 induced hypermethylation of the *Cldn1* gene (as SIRT1 deacetylates and also activates DNMT1), leading to downregulation of the tight junction protein Claudin-1 in podocytes, which protected against albuminuria [69]. In human mesangial cells, high glucose concentrations induced translocation of DNMT3a to the cytosol reducing its nuclear levels, thus facilitating *CTGF* hypo-methylation [70]. Indeed, increased *Trim13* promoter methylation contributed to downregulation of the expression of the E3 ubiquitin ligase TRIM13 in DKD glomeruli, which was associated to increased mesangial collagen synthesis [71]. DNA methylation may also contribute to modulate the expression of TGF-β1-regulated genes involved in the pathogenesis of DKD [60,72]. Indeed, reactive oxygen species (ROS) modulates DNA methylation [73] and through ROS-dependent DNA methylation of the *Tgfb1* locus contribute to mesangial fibrosis in DKD [74]. In kidneys of *db/db* mice and in mesangial cells cultured with high glucose, expression of the TET2 demethylase led to demethylation of the *TGFB1* promoter and increased TGF-β1 expression [75].

Inhibitors of DNMTs, such as 5-azacytidine (5-aza) and 5-aza-20-deoxycytidine (5-aza-2de, decitabine) induce DNA hypomethylation and may be beneficial in renal diseases [16,76] They reduced albuminuria in *db/db* mice [77] and 5-aza restored erythropoietin production in fibrotic murine kidneys, although it was not tested specifically in DKD [78]. In this regard, kidney fibrosis is a key contributor to DKD progression and TGF-β1-induced *KLF4* promoter hypermethylation and KLF4 downregulation in cultured human tubular cells was attenuated by decitabine [79], which also prevented high glucose-induced suppression of regulator of calcineurin 1 (RCAN1) expression in cultured podocytes [80]. RCAN1 has protective activity in podocytes. RCAN1 mRNA expression was suppressed in human and experimental DKD glomeruli and knockout of *Rcan1* aggravated albuminuria and podocyte injury in proteinuric mice [19]. Aberrant DNA methylation in peripheral immune cells could be also involved in DKD progression. DNMT1 is upregulated in peripheral immune cells from diabetic patients and correlated with the inflammatory response [81]. DNMT1 expression is also increased in immune cells from *db/db* mice and 5-aza treatment reduced the hypermethylation of negative regulators of mTOR activation, leading to mTOR pathway inactivation and reducing renal inflammation [81].

Additional compounds may influence DNA methylation indirectly, by modulating the expression and/or activity of DNMTs. Thus, at least part of the nephroprotective effect of BMP7 administration in diverse models of kidney fibrosis, including streptozotocin-induced DKD, was mediated by restoring TET3 mRNA expression and protein levels suppressed by TGF-β1, resulting in TET3-mediated restoration of the expression of the antifibrotic gene *Rasal1*. In this regard, aberrant *Rasal1* methylation and hydroxymethylation were corrected by BMP7 [82] which is a nephroprotective compound for different models of CKD, including diabetic nephropathy (DN) [83]. A potential mechanism of kidney protection may be the recovery of Klotho expression by reducing the characteristic Klotho promoter hypermethylation, which is observed in CKD [84]. This was related to a decrease in the expression of DNMT1/DNMT3a. Klotho is a kidney-derived protein with anti-ageing and nephroprotective properties [85]. 

Epigenetic changes can also contribute to discriminate between rapid progressors and those who will remain stable or assessing different response to treatment [62,86]. As an example, five DNA methylation (DNAm) sites differentiated diabetics treated and non-treated with statins in analysis of five cohort studies totaling 8270 patients in an epigenome-wide association study in blood. Two sites were associated with a glycemic trait or type 2 diabetes [87].

miRNAs can also regulate DNMTs. In tubular cells exposed to high glucose concentrations, miR-29b downregulation induced the expression of DNMTs resulting in upregulation of fibrotic genes, and this was alleviated by miR-29b mimics [88], while TGF-β1 enhanced DNMT1 and DNMT3a activity via inhibiting miR-152 and miR-30a in both renal cells and fibrotic (in this case non-diabetic) kidneys [76].

## 4. Histone Post-Translational Modifications in Diabetic Kidney Disease

Changes in histone post-translational modifications have been observed to exist and even to contribute to in diabetic kidney disease or kidney disease of diverse causes. 

### 4.1. Histone Methylation

Aberrant histone methylation has been observed in experimental and human DKD (Table 2) [89,90]. The first association between diabetes and altered histone methylation was observed in monocytes and lymphocytes from type 1 diabetic patients and in THP1 monocytes cultured with high glucose, where variations of histone methylation correlated with mRNA levels of targets genes [91,92,93]. 

The overall profile of histone methylation in DKD has not been fully characterized, but there is information on individual modifications or genes. In *db*/*db* mice kidneys, H3K4m2 (activating mark) are lower than in non-diabetic mice, and after uninephrectomy—which accelerates the progression of renal injury in *db/db* mice [94]—H3K4 methylation was enhanced [95]. Moreover, a CCL2 inhibitor, that prevents disease progression [96], also prevented the enhanced H3K4 methylation, suggesting that this epigenetic mark correlated with disease progression [95]. However, the effect of H3K4m2 on kidney gene expression is poorly understood. 

In two rodents models of type 1 diabetes, OVE26 mice and streptozotocin rats, the levels of H3K27m3, a repressive histone methylation mark, are reduced in key genes as *Mcp-1*, vimentin and the fibrosis marker *Fsp1*, while the levels of H3K4m2, an activating mark, are increased, suggesting that aberrant histone methylation may underlie differential kidney gene expression in DKD [97]. The histone demethylase KDM6A (also known as UTX) is overexpressed in OVE26 mice and may contribute to these observations [97]. The deleterious effect of histone demethylation in repressive marks has been also observed in cultured podocytes, since GSK-J4, a dual inhibitor of H3K27m3/2-demethylases, retrains the Notch pathway and favored podocyte differentiation (Figure 3) [98]. Furthermore, GSK-J4 attenuated renal injury in *db/db* mice [98]. Additionally, TGF-β1, a key promoter of fibrosis in DKD, increased the expression of histone demethylases JMJD3 and UTX and downregulated the EZH2 methyltranferase in mesangial cells [99]. In kidneys from streptozotocin rats and OVE26 mice, demethylases are upregulated, while the enrichment of EZH2 methylase and repressive methylation in pro-fibrotic genes is reduced. In human DKD, the kidney repressive histone mark H3K27m3 is also reduced and the expression of histone demethylase UTX is increased, supporting the role of histone demethylation of repressing sites in DKD [98]. Moreover, the inhibition of EZH2 in podocytes cultured under a high glucose environment and in streptozotocin diabetic rats decreased H3K27me marks at the *Pax6* promoter, activating PAX6 expression and promoting podocyte injury, oxidative stress and proteinuria [100]. SUV39H1, another histone methyltransferase of repressive mark H3K9m3, is also downregulated in kidneys from streptozotocin mice and in mesangial cells exposed to high glucose. Indeed, SUV39H1 overexpression decreased extracellular matrix production by mesangial cells [101,102]. 

SET7/9, a H3K4 mono-methyltransferase, mediates the TGF-β1-induced expression of pro-fibrotic genes since it was recruited to their promoters and SET7/9 siRNA targeting decreased extracellular matrix production induced by TGF-β1 in cultured mesangial cells [103]. Additionally, SET7/9 promotes the expression of inflammatory genes through histone methylation and transcription factor NF-κB recruitment in peripheral blood monocytes from streptozotocin diabetic mice and from type 2 diabetic patients, and in human aortic endothelial cells exposed to high glucose [104,105]. 

Interestingly, losartan, a representative AT1R blocker used to treat clinical DKD, partially reduced the permissive histone methylation observed in *db/db* mice, and this could explain the reduced expression of PAI-1, MCP-1 and RAGE under losartan treatment [106]. However, the effect of losartan over histone methylation of these genes was very weak, thus its overall contribution to improvement of DKD outcomes remains unclear [106]. 

Altogether, these data suggest that histone methylation could play a key role in altered gene expression during DKD, but functional in vivo studies specifically targeting individual enzymes are necessary to clarify their therapeutic target potential.

### 4.2. Histone Acetylation

There is evidence that histone acetylation contributes to DKD progression. Factors such as high glucose levels and diabetic complications can induce changes in the overall pattern of histone acetylation mediated by HATs and HDACs (Table 3) [107,108,109]. Furthermore, histone acetylation was implicated in EMT [110,111] and in the excessive kidney ECM deposition kidney [112].

TGF-β1, a key mediator of DKD, promotes histone acetylation. In cultured rat mesangial cells, TGF-β1 and high-glucose conditions activated p300/CBP, thus increasing H3K9/14ac at the fibronectin-1 (*Fn1*) promoter [113], and near Sp1 and Smad binding sites at the *Pai-1* and *p21* promoters [114,115], favoring their expression. Similarly, *Pai-1*, *Fn1* and *Ctgf* gene expression were also increased by activation of HAT p300/CBP and the presence of acetylated histones in their promoters in type 1 diabetic mice [113]. Additionally, TGF-β1 also increased acetylation of H3 (K9, 14, 27) and ETS-1 in glomeruli from diabetic *db/db* mice contributing to DKD through miR-192 expression [116]. H3K9/14ac is also present in *Ccl2 Rage* and *Pai-1* promoters under high glucose conditions, suggesting a regulatory function of H3K9/14ac in the expression of DKD-related genes [106]. p300/CBP also regulates the gene expression of collagen type I alpha 2 chain (*COL1A2*), an important extracellular matrix molecule, through modulation of histone acetylation at its promoter in dermal fibroblast and skin biopsies [117], and is also induced by TGF-β1 through p300/CBP recruitment in mouse mesangial cells [118].

Diabetes induces kidney histone acetylation, favoring proinflammatory gene expressions in rats [119] and in human blood monocytes through increased NF-κB activity and hyperacetylation of proinflammatory gene promoters [120]. In human and murine mesangial cells cultured under high glucose conditions and in kidneys from diabetic mice, H3K9 acetylation resulted in the increased expression of Thioredoxin-interacting protein (TXNIP), a key pathogenic factor in DKD [121].

Diabetic Akita mice, which have a point mutation in the Ins2 gene that leads to misfolding of insulin and type 1 diabetes, had increased H3K9 and H3K18 acetylation in renal cortex and this was decreased by apelin-13 treatment decreasing the expression of NF-κB inflammatory-related genes and this was associated with an increased expression of HDAC1 [122]. In blood monocytes from diabetic patients, there was enrichment in H3K9Ac promoters and H3K9Ac was associated to control of glycemia. Moreover, the top hyperacetylated promoters in diabetic patients were enriched in genes related to the NF-κB pathway and to diabetic complications [93].

Histone acetylation is a potential therapeutic target in DKD, by using HAT and HDAC inhibitors (Figure 3). HDAC1 is downregulated in renal cortex of Akita mice, and in rat glomerular mesangial cells cultured under high glucose conditions, resulting in histone hyperacetylation, which favors inflammatory gene expression [122]. HDAC inhibition lead to increased H3K9 acetylation and TGF-β1-induced gene expression, while HDAC1 and HDAC5 overexpression blocked TGF-β1-induced gene expression [114]. Similarly, increased activity of SIRT1, an important nephroprotective HDAC, by podocyte-specific SIRT1 overexpression or by the SIRT1 agonist BF175 treatment decreased podocyte injury and albuminuria in OVE26 mice [123]. In cultured podocytes, BF175 increased SIRT1-mediated activation of PGC-1α and protected against high glucose-mediated mitochondrial injury. In rat DKD, the HDAC inhibitors trichostatin A (TSA) and valproic acid (VPA) were protective. TSA blocked TGF-β1-induced extracellular matrix accumulation and increased the expression of E-cadherin in streptozotocin diabetic rats [124]. TSA is thought to increase E-cadherin expression through HDAC inhibition resulting in increased acetylation of its promoter, but it is unclear whether the effect over TGF-β1 expression depends on modulation of acetylation in nonhistone proteins [124]. VPA ameliorates renal injury in streptozotocin diabetic rats through the regulation of endoplasmic reticulum stress-associated proteins. VPA induces acetylation in the *Grp78* promoter and deacetylation in the C/EBP-homologous protein (*Chop*) promoter, resulting in an increased expression of GRP78 and a downregulation of CHOP [125]. The *Chop* promoter deacetylation seems to be mediated by ATF4 downregulation that is necessary for HAT binding at its promoter [125]. 

Curcumin prevents the development of renal injury in streptozotocin diabetic rats and this was associated with reduced levels of renal H3 acetylation [126]. Later, it was reported that the curcumin analog C66 inhibits HAT p300/CBP activity and consequently histone acetylation in streptozotocin diabetic mice preventing the expression of renal fibrotic genes [113]. C646, another p300/CBP inhibitor, inhibited TGF-β1-induced epithelial-mesenchymal transition in peritoneal mesothelial cells exposed to high glucose concentrations through modulation of H3 acetylation [127].

### 4.3. Other Histone Modifications

#### 4.3.1. Crotonylation

Constitutive histone crotonylation is present in different healthy tissues, including the kidney [20], and increased histone crotonylation has been described during experimental nephrotoxic AKI [43] (Table 3) suggesting a role of histone crotonylation in kidney injury, although there are not yet any studies in DKD [42]. In this regard, modulation of the levels of histone crotonylation modifies the outcome of kidney injury [43].

In murine tubular cells stimulated with the cytokine TWEAK, a mediator of kidney injury [128], and in kidneys from mice with AKI, histone crotonylation was increased and this was associated with decreased SIRT3 and PGC-1α expression and increased expression of the chemokine-encoding *Ccl2* gene [43]. In this regard, crotonate administration, which by increasing the substrate promoted histone crotonylation, increased kidney SIRT3 and PGC-1α expression in vivo and in cultured cells, while decreasing CCL2 expression and protecting from AKI [43]. These data point to a beneficial role of crotonate in renal injury, through increased histone crotonylation. However, studies assessing its role as therapeutic agent in DKD are required.

#### 4.3.2. β-Hydroxybutyrylation

Although few studies have explored the relationship between histone β-hydroxybutyrylation and DKD, there is evidence suggesting a potential beneficial effect. Despite the observation that increased serum BHB levels are associated with higher probability of death in hemodialysis patients regardless of the presence or absence of diabetic nephropathy [129], BHB suppresses oxidative stress and may be beneficial in DKD. In this regard, association does not mean causality. Thus, a ketogenic diet improved DKD and reduced oxidative stress genes in murine Type 1 (Akita) and Type 2 (*db/db*) diabetes [130]. It is thought that this effect is mediated by BHB, which protects against oxidative stress induced by glucose in neuronal cells, but this was not tested in renal cells [130]. In human embryonic kidney cells, histone Kbhb is regulated by BHB levels while histone acetylation was not. This may have a physiological relevance in DKD, since serum BHB levels are increased in both fasted mice and streptozotocin diabetic mice [47]. Indeed, higher serum BHB levels are associated with increased histone Kbhb levels in both liver of streptozotocin diabetic mice and in kidney of fasted mice, and histone Kbhb had a role in reprograming gene expression to adapt cells to changes in energy sources (Table 3) [47]. BHB inhibition of HDACs may improve the metabolic profile and redox state by inducing oxidative stress resistance through the expression of FOXO3A and MT2 in murine kidney [45]. Concerning DN, post-treatment with sodium butyrate (NaB) in streptozotocin rats was nephroprotective and reduced HDAC activity suggesting that this protection was mediated by modulate acetylation of histones [131]. Nevertheless, further studies are necessary to further understand the global and local impact of histone Kbhb on DKD and its therapeutic potential. 

### 4.4. Epigenetic Reader Modifiers

Selective BET inhibitors (iBETs) block the interaction between the bromodomain on BET proteins and acetylated proteins [136,137]. In cell culture, BRD4 inhibition by small interfering RNA or by pharmacological iBETs downregulates proinflammatory and profibrotic gene expression [136,137,138,139]. Transcription factors also contain acetylated residues. The RelA subunit of the proinflammatory transcription factor NF-κB can be acetylated in Lys310 leading to activation. Studies on cancer have described that BRD4 binding to acetylated Lys310 of RelA is essential to activate specific NF-κB target genes [140,141,142]. BET inhibition with JQ1 reduced RelA nuclear levels in several experimental models of renal damage and in TNF-α-exposed kidney cells, thereby blocking NF-κB transcriptional activation and downregulating several NF-κB-controlled genes, including *Ccl2* and *Il17a* [143]. Interestingly, all the mentioned factors, TNF-α, NF-κB, CCL-2 and IL-17A, contribute to the pathogenesis of DKD [144,145].

iBETs have been beneficial in diverse experimental diseases, including malignancy, infections, autoimmunity, inflammation and fibrotic disorders [136,137,139] 25407682. Specifically, BET inhibition also protected from diabetes and improved renal function and was nephroprotective in experimental kidney disease [136,143]. 

iBET-762 prevented diabetes in female nonobese diabetic (NOD) mice, a model of type 1 diabetes [146]. In this regard, BET inhibition in pancreatic β-cells increased insulin secretion [147], suggesting that these drugs may be useful to treat insulin resistant/diabetic patients. BRD4 also modulates the induction of a senescence-associated secretory phenotype in islet cells [146]. Moreover, BET proteins regulate pancreatic development [148], and diabetic intervertebral disc degeneration [149]. Related to diabetes-induced tissue injury, in streptozotocin-induced diabetic mice, JQ1 suppressed cardiac fibrosis and improved cardiac function by modulating Caveolin-1/TGF-β1 signaling in cardio fibroblasts and inhibiting cardiomyocyte apoptosis [150]. BRD4 is also involved in high glucose-induced cardiomyocyte hypertrophy through the AKT pathway [151]. Less information is available for the kidney. However, the results are also consistent with nephroprotection by BET inhibition. Thus, in cultured podocytes, BRD4 gene silencing or JQ1 inhibited high glucose-induced podocyte injury, whereas BRD4 overexpression induced apoptosis, but it is unknown if this is mediated by modification in histone binding [152]. 

## 5. Relationship of Epigenetic Modifications to Key Pathogenic Processes in DKD 

The information on epigenetic modulation and DKD can be summarized in terms of potential contribution to specific pathogenic processes, such as podocyte injury, inflammation and fibrosis through the modulation of gene transcription in kidney cells and/or leukocytes (Table 4). Despite these studies, it is difficult to pinpoint the effect of a certain histone post-translational modifications or DNA methylation to a specific cell type or a specific molecule or group of molecules, given that uncharacterized effects in other cell types or other genes may be contribute to the observed phenotype. For this very same reason, it is very difficult to clarify whether the relationship between certain epigenetic modifications with DKD features represent associations versus causation. If no interventional studies were performed, it is impossible to differentiate association from causation. However, even if interventional studies were performed, it would be unclear whether the observed changes in gene expression in cultured cells driven by promoting or inhibiting a certain epigenetic feature are the key drivers of any potential in vivo therapeutic effect or whether they may represent epiphenomena and the in vivo effect is driven by a well-orchestrated response in which the gene analyzed played only a minor role or no role at all. Alternatively, the key cell driving the in vivo response may not even be a renal cell. Thus, despite observing an impact of an epigenetic modification on cultured podocytes, the key cell for kidney protection in vivo may be a leukocyte subtype. This is the case even when well characterized mediators of DKD such as TGFβ1 are shown to be modulated both in culture and in vivo. This issue will only be addressed when single cell epigenetic techniques are developed and combed with currently available single cell transcriptomics data.

## 6. Epigenetic Modifiers as Therapeutic Agents or Targets in Clinical Diabetic Kidney Disease

Clinical research into epigenetics and epigenetic modifiers is increasing. According to Clinicaltrials.gov, there are at least 290 clinical studies on the topic (https://clinicaltrials.gov/ct2/results?cond=epigenetic&term=&cntry=&state=&city=&dist=&Search=Search; accessed on April 17 2020). Most studies focus on epigenetics in hematological diseases, cancer, autoimmune and inflammatory disease and cardiovascular risk factors, as diabetes, obesity and atherosclerosis and include testing of iBETs, mostly in malignancy [136]. Indeed, 55 studies are ongoing in field of diabetes, most of them not testing direct epigenetic modifiers. 

The BD2 selective inhibitor apabetalone (RVX-208/RVX000222) was evaluated in patients with type 2 DM and high cardiovascular risk (https://ClinicalTrials.gov/NCT02586155). It is the epigenetic modifier with most advanced clinical development in diabetes and cardiovascular and kidney disease, as phase-3 results were recently reported, as discussed below). Apabetalone modulates the expression of a large variety of genes, including complement and coagulations factors, cardiovascular disease markers, C-reactive protein and, of specific interest for nephrologists, alkaline phosphatase, and cholesterol transport genes.

The phase IIb SUSTAIN and ASSURE trials assessed the effect of apabetalone on cardiovascular events in high-risk diabetic patients with coronary artery disease. A post-hoc sub-analysis [153] of patients with eGFR <60 mL/min/1.73 m^2^ disclosed that apabetalone patients had a significant reduction (*p* = 0.02) of alkaline phosphatase levels of −14% compared to −6% in the placebo group. Alkaline phosphatase levels are a risk factor for mortality in CKD patients and are correlated and are thought to contribute to vascular calcification and inflammation, and also to cardiovascular events [154,155]. A further multi-center clinical trial (NCT03160430) will compare, in a sequential cross-over study with four weeks washout in between, the impact of six weeks of apabetalone (100 mg/12 h) or placebo on plasma alkaline phosphatase in end stage renal disease patients receiving hemodialysis.

A phase-3 clinical trial, BETonMACE, was recently completed [156,157]. It enrolled 2425 patients with recent acute coronary syndrome, type 2 diabetes and low HDL cholesterol on statins to apabetalone 100 mg/12 h or placebo for 120 weeks. The trial failed to meet the primary endpoint of cardiovascular death, myocardial infarction, or stroke [157]. During a median follow-up of 26.5 months, 274 primary end points occurred: 125 (10.3%) in apabetalone-treated patients and 149 (12.4%) in placebo-treated patients (hazard ratio, 0.82 [95% CI, 0.65–1.04]; *p* = 0.11). In a prespecified sensitivity analysis that included adjudicated cardiovascular deaths but excluded deaths of undetermined cause, the primary end point was still not met (HR, 0.79; 95% CI, 0.62–1.01; *p* = 0.06). Although there was a predefined secondary endpoint of change in kidney function in patients with eGFR <60 mL/min/1.73 m^2^, formal statistical testing of the key secondary end points and prespecified subgroups was not performed as this was precluded by the prespecified analysis plan once the primary endpoint was not met. In any case, an exploratory analysis of secondary end points suggested a reduced risk of congestive heart failure hospitalizations. However, no impact on inflammation, as assessed by C reactive protein levels, was observed. More patients were allocated to apabetalone than the placebo discontinued study drug (114 [9.4%] vs. 69 [5.7%]), for reasons including elevations of liver enzyme levels (35 [2.9%] vs. 11 [0.9%]), raising safety concerns [157]. The incidence of alanine aminotransferase elevation exceeding five times the upper limit of normal was 4.7-fold higher in the apabetalone than in the placebo groups, but this was fully reversible and Hy´s law thresholds for liver toxicity were not met in any patient. Nausea occurred more often with apabetalone vs. placebo (26 [2.1%] vs. 7 [0.6%]), raising issue of tolerability of the drug.

Six apabetalone clinical trials are evaluating its safety, pharmacokinetics and pharmacodynamics, and two are focused on chronic kidney disease. A Phase-1 and -2 trial (NCT03228940) will evaluate the safety and effect on key biomarkers (e.g., markers of inflammation, CKD-MBD and glycolipid storage) of apabetalone 100 mg/12 h for 16 weeks in Fabry disease patients. Fabry Disease is a genetic X-linked disorder of lysosomal storage caused by mutations in the *GLA* gene leading to accumulation glycolipids and kidney and heart disease, which shares pathogenic pathways with DKD [158,159]. The trial is expected to be completed by the end of 2020.

Two other interventional clinical studies (NCT03817749, NCT04194450) in pre-diabetic patients are assessing, as a secondary endpoint, whether monocyte H3 acetylation at Lys9 and Lys14 changes in response to oral ketone supplements (a ketone ester and (R)-3-hydroxybutyl (R)-3-hydroxybutyrate, respectively) for 14 days. β-hydroxybutyrate increases in individuals treated with SGLT2 inhibitors, as discussed below. The short-chain fatty acid butyrate, a product generated by the gut microbiota can be converted to β-hydroxybutyrate [160]. Butyrate decreases proteinuria in diabetic rats [131]. Since an altered microbiota characterized by decreased butyrate-producing bacteria has been described in diabetes, an ongoing RCT (NCT04073927) in type 1 DM patients with DKD is exploring the impact of 3.6 g/day oral sodium butyrate or placebo for 12 weeks and has albuminuria and GFR as secondary endpoints.

Regarding safety and specific populations that may benefit form epigenetic interventions, there is not yet sufficient information, as interventions targeting epigenetic modifications in DKD are still at the clinical trial stage

## 7. SGLT2 Inhibitors and Epigenetics

Given the unexpected beneficial impact of SGLT2 inhibitors on cardiac and kidney outcomes in diabetes, DKD and heart failure, there is a considerable debate on which may be the molecular mechanisms involved [13]. This may be related to a tubular-mediated hemodynamic impact, to protection of tubular cells form excess glucose or to other metabolic effects, such as increased ketone levels [13,161,162,163]. Given the increasing information on the role of epigenetic regulation in DKD, there is the distinct possibility that SGLT2 inhibitors may indirectly regulate DNA methylation and/or histone post-translational modifications. A PubMed search on 23 April 2020 for (methylation OR acetylation OR crotonylation OR histone) AND (SGLT2 OR dapagliflozin OR canagliflozin OR empagliflozin) did not disclose any relevant information. We suggest that this is a fertile field of exploration both to understand the molecular mechanisms of the beneficial effects of SGLT2 inhibitors as well as to further understand the role of epigenetic regulation in DKD. In this regard, SGLT2 inhibitors increase plasma and tissue levels of the ketone 3-hydroxybutyric acid, which induced β-hydroxybutyrylation of H3 at Lys9 of the adiponectin gene in adipocytes independent of their acetylation or methylation, identifying a new potential histone post-translational modification relevant to the therapeutic effect of SGLT2 inhibitors [52]. Despite the scarce information on DNA methylation and histone epigenetics, there is already some information on miRNAs. In an open label study in 40 diabetic patients comparing treatment with dapagliflozin or thiazides, circulating miR30e-5p was upregulated and miR199a-3p downregulated in dapagliflozin-treated patients [164]. This study suggests the indeed SGLT2 inhibitors may modulate epigenetic regulators.

## 8. Summary and Future Perspectives

The concept of the epigenetic regulation of gene expression has evolved from a fixed-at-birth-or-early-development feature to a dynamic characteristic that may be modified in response to the environment or therapeutic modulation. Thus, tools are available to activate or inhibit the enzymes involved in attaching or detaching the epigenetic marks, as well as to interfere with readers of epigenetic information. Interference with the BET reader proteins is already undergoing clinical trials in the DKD field. Additionally, the substrate availability regulates histone post-translational modifications, as demonstrated for histone crotonylation or β-hydroxybutyrylation. These tools have been used to demonstrate nephroprotective effects of therapeutically targeting epigenetic regulation in diverse nephropathies, including DKD. However, some maneuvers remain underexplored in the DKD field, such as the nephroprotective role of an overall increase in kidney histone crotonylation as observed in non-diabetic kidney injury. Finally, commonly used drugs may indirectly regulate epigenetic mechanisms. The most unexplored are the epigenetic consequences of prescribing SGLT2 inhibitors. There is an urgent need to do so, given that their cardio- and nephroprotective potential far exceeds expectations and is currently not well explained based on our understanding of their mechanisms of action. A role for an indirect modulation of epigenetics is clearly in need of further studies, given the impact of SGLT2 inhibitors on 3-hydroxybutyric acid, a driver of histone β-hydroxybutyrylation. Interestingly, a low-calorie intake is the only maneuver that consistently increased lifespan in all species tested and it is thought that fasting is a key contributor to this effect. Fasting also increases 3-hydroxybutyric acid levels, increasing the histone β-hydroxybutyrylation of, for example, the *Ppargc1a* gene encoding PGC-1α [50], which is a key nephroprotective molecule [135,165,166,167]. Finally, despite the description of the association of some epigenetic modifications of specific features, and even to specific molecules involved in DKD, there is an insufficient understanding on the cause-and-effect relationship between these specific molecular changes and the overall impact of targeting epigenetic modifications in vivo. In this regard, given that interventions targeting epigenetic modifications in DKD are still at the clinical trial stage, it is not yet known whether early intervention in these pathways can prevent disease or whether later intervention will reverse established diseases in humans.

## Figures and Tables

**Table 1:** Aberrant DNA-methylation in diabetic nephropathy (DN) and cells cultured under high glucose (HG) conditions.

Change in DN *	Effect in Target Gene **	Model	Cell or Tissue	Reference
**↓ DNA-methylation**	↑ *MIOX*	STZ mice	Kidney	[64]
		HG in human cells	Tubular (HK-2)	
	↑ *MMP9*	HG in human cells	Podocytes	[66]
	↑ *CLDN-1*	STZ mice	Kidney	[69]
		HG in human cells	Renal epithelial cells	
	↑ *CTGF*	HG in human cells	Mesangial cells	[70]
	↑ *TGFB1*	*db/db* mice	Mesangial cells from diabetic mice	[74,75]
		*db/db* mice	Kidney	
		HG in human cells	Mesangial cells	
	↑ *Agt, Abcc4, Slco1a1*	*db/db* mice	Proximal tubules	[63]
**↑ DNA-methylation**	↓ mTOR upstream inhibitors	*db/db* mice	Immune cells from diabetic mice	[81]
	↓ *NPHS1*	*db/db* mice	Kidney	[67,68]
		STZ and *db/db* mice	Kidney and isolated podocytes	
		HG in human cells	Podocytes	
	↓ *Trim13*	STZ and *db/db* mice	Kidney	[71]
	↓ *KLF4*	TGF-β1 in human cells	HK-2	[79]
	↓ *RCAN1*	HG in human cells	Podocytes	[80]
	↓ *Rasal1*	STZ mice	Kidney	[82]
	↓ *ESX1, GRIA3*	HG in human cells	Podocytes	[65]

Streptozotocin (STZ) induces insulin-deficient diabetes that resembles type 1 DM, although there is no autoimmune component, while *db/db* mice are a model for type 2 DM. * Downward looking arrows mean decreased DNA-methylation and upward looking arrows mean increased DNA-methylation. ** Upward looking arrows mean increased gene expression and downward looking arrows mean decreased gene expression.

**Table 2:** Altered histone methylation in diabetic nephropathy (DN) and cells cultured under high glucose (HG) conditions.

Histone Methylation		DN	Model	Sample	Reference
**Activating marks**	H3K4m2	↓ global	*db/db* mice (early time points)	Kidney	[95]
		↑ global	Uninephrectomiced *db/db* mice		
		↑ in *Ccl21, Fsp1*	OVE26 mice (T1D)	Kidney	[97]
			STZ rats	Kidney	
	H3K4m1/2/3	↑ in EMT-associated genes	HG in rat cells	Mesangial cells	[103]
	H3K4m3	↑ global	T1D patients	Blood monocytes	[93]
**Repressive mark**	H3K9m2	↑ in *IL1A*	HG in human cells	THP-1 monocytes	[91]
			T1D and T2D patients	Blood monocytes	
		↑ in *CLTA4*	T1D patients	Blood lymphocytes	[92]
	H3K9m3	↓ in *Fn1, p21*	HG in mouse cells	Mesangial cells	[101]
	H3K9m2/3	↓ in EMT-associated genes	HG in rat cells	Mesangial cells	[103]
	H3K27m2	↓ global	Adriamycin mice	Isolated podocytes	[98]
			DKD patients	Isolated podocytes	
		↓ in *Pai-1, Ccl2*	STZ rats	Kidney	[99]
			TGF-β1 in rat cells	Mesangial cells	
		↓ in *Pax6*	STZ rats	Kidney	[100]
			HG in mouse cells	Podocytes	
	H3K27m3	↓ in *Ccl21, Fsp1*	OVE26 mice (T1D)	Kidney	[97]
			STZ rats	Kidney	

Streptozotocin (STZ) induces insulin-deficient diabetes that resembles type 1 DM, although there is no autoimmune component while *db/db* mice are a model for type 2 DM (T2D). Upward looking arrows mean increased methylation and downward looking arrows mean decreased methylation.

**Table 3:** Altered histone acetylation, β-hydroxybutyrylation and crotonylation in diabetic nephropathy (DN) and cells cultured under high glucose (HG) conditions. Streptozotocin (STZ) induces insulin-deficient diabetes that resembles type 1 DM, although there is no autoimmune component while *db/db* mice are a model for type 2 DM (T2D).

Histone Modification	Change in DN *	Effect in Target Gene **	Model	Sample	Ref.
**Acetylation**	↑ H2BK5Ac	↑ *Mme*	STZ rats	Kidney	[119]
	↑ H3K9Ac	Global	Uninephrectomiced *db/db* mice	Kidney	[95]
			Akita mice (T1D)	Kidney	[122]
			HG in rat cells	Mesangial	
			T1D patients	Blood monocytes	[93]
		↑ *TXNIP*	Sur1-E1506K^+/+^ mice (T2D)	Kidney	[121]
			HG in human, murine cells	Mesangial	
	↑ H3K9/14Ac	*↑ Pai-1 and p21*	HG in rat cells	Mesangial	[114]
		*↑ Fn1, Ctgf, Pai-1*	STZ mice	Kidney	[113]
		*↑ Ets1*	*db/db* mice	Kidney	[116]
		*↑ TNF, COX2*	HG in human cells	THP-1 monocytes	[120]
	↑ H3K18Ac	↑ global	Akita mice (T1D)	Kidney	[122]
			HG in rat cells	Mesangial	
		↑ *Mme*	STZ rats	Kidney	[119]
	↑ H3K23Ac	↓ global	*db/db* mice	Kidney	[95]
		↑ global	Uninephrectomiced *db/db* mice	Kidney	
	↑ H4Ac	↑ *Grp78, Chop Atf4*	STZ in rats	Kidney	[125]
	↑ H4K5/8/12Ac	↑ *TNF, COX2*	HG in human cells	THP-1 monocytes	[120]
**Crotony-lation**	N/A (increased, global and *Ppargc1a and Sirt3 genes* in AKI)	N/A (Crotonylation increased *PGC-1α* and *SIRT3*, and decreased *CCL2* expression ***)	N/A (AKI mice)	N/A (Kidney and tubular cells)	N/A [43]
**β-hydroxy-butyrylation**	↑ H3K9bhb	Global	STZ mice	Liver	[47]
			Fasted mice	Kidney	
	↑ H3K18bhb	Global	STZ mice	Liver	
	↑ H4K8bhb	Global	Fasted mice	Kidney	

* Upward looking arrows mean increased histone acetylation. ** Upward looking arrows mean increased gene expression. *** *CCL2* promotes and *SIRT3* and *PGC-1a* protect from experimental DN [132,133,134,135]. N/A: data for DN not available.

**Table 4:** Epigenetic modulation and relationship to key pathogenic processes in diabetic nephropathy.

Injury	DNA or Protein Modification	Effect in Target Genes	Relation between Epigenetic Modification and Gene Target *	Sample/Model or Treatment	Ref.
Podocyte injury	↑ DNA-methylation	*↓ GRIA3*	Causal	Human and murine podocytes/HG	[65]
	↑ DNA-methylation	↓ *Nphs1*	Association	Kidney/db/db mice Kidney/STZ rat and db/db mice	[67] [68]
	↓ H3K27m2	↑ *Pax6*	Causal	Murine podocytes/HG Kidney/STZ rats	[100]
Inflammation	↑ DNA-methylation	*↓ negative regulators of mTOR*	Association	PBMCs/db/db mice	[81]
	↓ H3K27m2	↑ *CCL2*	Association	Rat mesangial cells/ TGF-β1 Kidney/OVE26 mice (T1D)	[99] [97]
	↑ H3K4 m1/m2/m3	↑ *inflammatory genes* *↑ inflammatory genes* *↑ Mcp-1*	Causal Association Causal	Macrohages/diabetic mice Monocytes /diabetic patients Kidney/OVE26 mice (T1D)	[104] [105] [97]
	↑ H2BK5ac	*↑ Mme*	Association	Kidney/STZ rats	[119]
	↑ H3K18ac				
	↑ H3K9/14ac	↑ *TNFα and COX-1*	Association	Monocytes/Diabetic human	[120]
		*↑ Txnip*	Association	Kidney/Diabetic mice	[121]
				Human and mouse mesangial cells/HG	
		*↑ Mcp-1*	Association	Kidney/db/db mice Murine mesangial cells/HG	[106]
	↑ H4K5/8/12ac	*↑ TNFα and COX-1*	Association	THP-1 monocytes/ HG	[120]
Fibrosis ↑ EMT	↓ DNA-methylation	↑ *MMP9*	Association	Human podocytes/HG Kidney/STZ rat	[66]
	↑ DNA-methylation	*↓ KLF4*	Causal	Human proximal tubular cells/ TGF-β1	[79]
	↑ H3K4m2	*↑ Fsp1*	Association	Kidney/STZ rats	[97]
	↓ H3K27m3				
Fibrosis ↑ ECM	↑ DNA-methylation	*↓ Trim13*	Causal	Kidney/STZ rat and db/db mice	[71]
	↓ DNA-methylation	↑ *MIOX*	Association	Human proximal tubular cells/HG Kidney/STZ rat	[64]
	↓ H3K9m3	*↑ Fn-1, p21*	Causal	Murine mesangial cells/HG	[101]
	↑ H3K4m1/2/3 ↓ H3K9m2/3	*↑ ECM-associated genes*	Causal Association	Rat mesangial cell/HG	[103]
	↑ H3K9/14ac	*↑ Fn-1 and Pai-1*	Association	Kidney/STZ-induced diabetic mice	[113]
		*↑ Pai-1 and p21*	Association	Rat mesangial cells/HG	[114,115]
		*↑ Pai-1 and Rage*	Causal	Kideny/db/db mice Murine mesangial cells/HG	[106]
	↑ H4Kac	*↑ Col1A2*	Causal	Murine mesangial cells/TGF-β	[118]
Fibrosis (TGFβ1)	↓ DNA-methylation	*↑ Tgf-β1*	Association	Kidney/db/db mice	[74]
			Causal	Kidney/db/db mice Human mesangial cells/HG	[75]

* Association: Changes in epigenetic markers was associated with changes in gene expression. Causal: potentially causal as induction of changes in epigenetic markers was followed by changes in gene expression and both increased and decreased epigenetic markers had coherent impact on gene expression. HG: high glucose concentration. PBMC: peripheral blood mononuclear cells.

## References

[1] Bikbov B., Purcell C.A., Levey A.S., Smith M., Abdoli A., Abebe M., Adebayo O.M., Afarideh M., Agarwal S.K., Agudelo-Botero M. (2020). Global, regional, and national burden of chronic kidney disease, 1990–2017: A systematic analysis for the Global Burden of Disease Study 2017. Lancet.

[2] The Lancet (2018). GBD 2017: A fragile world. Lancet.

[3] Thomas B. (2019). The Global Burden of Diabetic Kidney Disease: Time Trends and Gender Gaps. Curr. Diabetes Rep..

[4] Foreman K.J., Marquez N., Dolgert A., Fukutaki K., Fullman N., McGaughey M., Pletcher M.A., Smith A.E., Tang K., Yuan C.W. (2018). Forecasting life expectancy, years of life lost, and all-cause and cause-specific mortality for 250 causes of death: Reference and alternative scenarios for 2016-40 for 195 countries and territories. Lancet.

[5] Fernandez-Fernandez B., Fernandez-Prado R., Górriz J.L., Martinez-Castelao A., Navarro-González J.F., Porrini E., Soler M.J., Ortiz A. (2019). Canagliflozin and Renal Events in Diabetes with Established Nephropathy Clinical Evaluation and Study of Diabetic Nephropathy with Atrasentan: What was learned about the treatment of diabetic kidney disease with canagliflozin and atrasentan?. Clin. Kidney J..

[6] Perez-Gomez M.V., Sanchez-Niño M.D., Sanz A.B., Martín-Cleary C., Ruiz-Ortega M., Egido J., Navarro-González J.F., Ortiz A., Fernandez-Fernandez B. (2015). Horizon 2020 in Diabetic Kidney Disease: The Clinical Trial Pipeline for Add-On Therapies on Top of Renin Angiotensin System Blockade. J. Clin. Med..

[7] Sanchez-Niño M.D., Sanz A.B., Sanchez-Lopez E., Ruiz-Ortega M., Benito-Martin A., Saleem M.A., Mathieson P.W., Mezzano S., Egido J., Ortiz A. (2012). HSP27/HSPB1 as an adaptive podocyte antiapoptotic protein activated by high glucose and angiotensin II. Lab. Investig..

[8] Sanchez-Niño M.D., Sanz A.B., Ihalmo P., Lassila M., Holthofer H., Mezzano S., Aros C., Groop P.H., Saleem M.A., Mathieson P.W. (2009). The MIF receptor CD74 in diabetic podocyte injury. J. Am. Soc. Nephrol..

[9] Navarro-González J.F., Sánchez-Niño M.D., Donate-Correa J., Martín-Núñez E., Ferri C., Pérez-Delgado N., Górriz J.L., Martínez-Castelao A., Ortiz A., Mora-Fernández C. (2018). Effects of Pentoxifylline on Soluble Klotho Concentrations and Renal Tubular Cell Expression in Diabetic Kidney Disease. Diabetes Care.

[10] Fernandez-Fernandez B., Izquierdo M.C., Valiño-Rivas L., Nastou D., Sanz A.B., Ortiz A., Sanchez-Niño M.D. (2018). Albumin downregulates Klotho in tubular cells. Nephrol. Dial. Transplant..

[11] Sanchez-Niño M.D., Sanz A.B., Lorz C., Gnirke A., Rastaldi M.P., Nair V., Egido J., Ruiz-Ortega M., Kretzler M., Ortiz A. (2010). BASP1 promotes apoptosis in diabetic nephropathy. J. Am. Soc. Nephrol..

[12] Sanchez-Niño M.D., Bozic M., Córdoba-Lanús E., Valcheva P., Gracia O., Ibarz M., Fernandez E., Navarro-Gonzalez J.F., Ortiz A., Valdivielso J.M. (2012). Beyond proteinuria: VDR activation reduces renal inflammation in experimental diabetic nephropathy. Am. J. Physiol.-Ren. Physiol..

[13] Sarafidis P., Ferro C.J., Morales E., Ortiz A., Malyszko J., Hojs R., Khazim K., Ekart R., Valdivielso J., Fouque D. (2019). SGLT-2 inhibitors and GLP-1 receptor agonists for nephroprotection and cardioprotection in patients with diabetes mellitus and chronic kidney disease. A consensus statement by the EURECA-m and the DIABESITY working groups of the ERA-EDTA. Nephrol. Dial. Transplant..

[14] Perkovic V., Jardine M.J., Neal B., Bompoint S., Heerspink H.J., Charytan D.M., Edwards R., Agarwal R., Bakris G., Bull S. (2019). Canagliflozin and Renal Outcomes in Type 2 Diabetes and Nephropathy. N. Engl. J. Med..

[15] Association A.D. (2019). 11. Microvascular Complications and Foot Care in Diabetes—2019. Diabetes Care.

[16] Fontecha-Barriuso M., Martin-Sanchez D., Ruiz-Andres O., Poveda J., Sanchez-Niño M.D., Valino-Rivas L., Ruiz-Ortega M., Ortiz A., Sanz A.B. (2018). Targeting epigenetic DNA and histone modifications to treat kidney disease. Nephrol. Dial. Transplant..

[17] Ruiz-Andres O., Sanchez-Niño M.D., Moreno J.A., Ruiz-Ortega M., Ramos A.M., Sanz A.B., Ortiz A. (2016). Downregulation of kidney protective factors by inflammation: Role of transcription factors and epigenetic mechanisms. Am. J. Physiol.-Ren. Physiol..

[18] Zhang D., Tang Z., Huang H., Zhou G., Cui C., Weng Y., Liu W., Kim S., Lee S. (2019). Metabolic regulation of gene expression by histone lactylation. Nature.

[19] Susztak K. (2014). Understanding the epigenetic syntax for the genetic alphabet in the kidney. J. Am. Soc. Nephrol..

[20] Tan M., Luo H., Lee S., Jin F., Yang J.S., Montellier E., Buchou T., Cheng Z., Rousseaux S., Rajagopal N. (2011). Identification of 67 histone marks and histone lysine crotonylation as a new type of histone modification. Cell.

[21] Jin S.G., Wu X., Li A.X., Pfeifer G.P. (2011). Genomic mapping of 5-hydroxymethylcytosine in the human brain. Nucleic Acids Res..

[22] Li L.X., Agborbesong E., Zhang L., Li X. (2019). Investigation of epigenetics in kidney cell biology. Methods Cell Biol..

[23] Beckerman P., Ko Y.A., Susztak K. (2014). Epigenetics: A new way to look at kidney diseases. Nephrol. Dial. Transplant..

[24] Bomsztyk K., Denisenko O., Wang Y. (2018). DNA methylation yields epigenetic clues into the diabetic nephropathy of Pima Indians. Kidney Int..

[25] Liao J., Karnik R., Gu H., Ziller M.J., Clement K., Tsankov A.M., Akopian V., Gifford C.A., Donaghey J., Galonska C. (2015). Targeted disruption of DNMT1, DNMT3A and DNMT3B in human embryonic stem cells. Nat. Genet..

[26] Ko M., An J., Pastor W.A., Koralov S.B., Rajewsky K., Rao A. (2015). TET proteins and 5-methylcytosine oxidation in hematological cancers. Immunol. Rev..

[27] Audia J.E., Campbell R.M. (2016). Histone Modifications and Cancer. Cold Spring Harb. Perspect. Biol..

[28] Blackshaw L.A., Grundy D. (1989). Responses of vagal efferent fibres to stimulation of gastric mechano- and chemoreceptors in the anaesthetized ferret. J. Auton. Nerv. Syst..

[29] Black J.C., Whetstine J.R. (2013). Tipping the lysine methylation balance in disease. Biopolymers.

[30] Greer E.L., Shi Y. (2012). Histone methylation: A dynamic mark in health, disease and inheritance. Nat. Rev. Genet..

[31] Shi Y., Lan F., Matson C., Mulligan P., Whetstine J.R., Cole P.A., Casero R.A., Shi Y. (2004). Histone demethylation mediated by the nuclear amine oxidase homolog LSD1. Cell.

[32] Ramakrishnan S., Pili R. (2013). Histone deacetylase inhibitors and epigenetic modifications as a novel strategy in renal cell carcinoma. Cancer J..

[33] Davey C.A., Sargent D.F., Luger K., Maeder A.W., Richmond T.J. (2002). Solvent mediated interactions in the structure of the nucleosome core particle at 1.9 a resolution. J. Mol. Biol..

[34] Kouzarides T. (2007). Chromatin modifications and their function. Cell.

[35] Dokmanovic M., Marks P.A. (2005). Prospects: Histone deacetylase inhibitors. J. Cell. Biochem..

[36] Wei W., Mao A., Tang B., Zeng Q., Gao S., Liu X., Lu L., Li W., Du J.X., Li J. (2017). Large-Scale Identification of Protein Crotonylation Reveals Its Role in Multiple Cellular Functions. J. Proteom. Res..

[37] Fellows R., Denizot J., Stellato C., Cuomo A., Jain P., Stoyanova E., Balázsi S., Hajnády Z., Liebert A., Kazakevych J. (2018). Microbiota derived short chain fatty acids promote histone crotonylation in the colon through histone deacetylases. Nat. Commun..

[38] Sabari B.R., Tang Z., Huang H., Yong-Gonzalez V., Molina H., Kong H.E., Dai L., Shimada M., Cross J.R., Zhao Y. (2015). Intracellular crotonyl-CoA stimulates transcription through p300-catalyzed histone crotonylation. Mol. Cell.

[39] Liu X., Wei W., Liu Y., Yang X., Wu J., Zhang Y., Zhang Q., Shi T., Du J.X., Zhao Y. (2017). MOF as an evolutionarily conserved histone crotonyltransferase and transcriptional activation by histone acetyltransferase-deficient and crotonyltransferase-competent CBP/p300. Cell Discov..

[40] Wei W., Liu X., Chen J., Gao S., Lu L., Zhang H., Ding G., Wang Z., Chen Z., Shi T. (2017). Class I histone deacetylases are major histone decrotonylases: Evidence for critical and broad function of histone crotonylation in transcription. Cell Res..

[41] Rousseaux S., Khochbin S. (2015). Histone Acylation beyond Acetylation: Terra Incognita in Chromatin Biology. Cell J..

[42] Martinez-Moreno J.M., Fontecha-Barriuso M., Martín-Sánchez D., Sánchez-Niño M.D., Ruiz-Ortega M., Sanz A.B., Ortiz A. (2020). The Contribution of Histone Crotonylation to Tissue Health and Disease: Focus on Kidney Health. Front. Pharmacol..

[43] Ruiz-Andres O., Sanchez-Niño M.D., Cannata-Ortiz P., Ruiz-Ortega M., Egido J., Ortiz A., Sanz A.B. (2016). Histone lysine crotonylation during acute kidney injury in mice. Dis. Models Mech..

[44] Anson R.M., Guo Z., de Cabo R., Iyun T., Rios M., Hagepanos A., Ingram D.K., Lane M.A., Mattson M.P. (2003). Intermittent fasting dissociates beneficial effects of dietary restriction on glucose metabolism and neuronal resistance to injury from calorie intake. Proc. Natl. Acad. Sci. USA.

[45] Shimazu T., Hirschey M.D., Newman J., He W., Shirakawa K., Le Moan N., Grueter C.A., Lim H., Saunders L.R., Stevens R.D. (2013). Suppression of oxidative stress by β-hydroxybutyrate, an endogenous histone deacetylase inhibitor. Science.

[46] Bae H.R., Kim D.H., Park M.H., Lee B., Kim M.J., Lee E.K., Chung K.W., Kim S.M., Im D.S., Chung H.Y. (2016). β-Hydroxybutyrate suppresses inflammasome formation by ameliorating endoplasmic reticulum stress via AMPK activation. Oncotarget.

[47] Xie Z., Zhang D., Chung D., Tang Z., Huang H., Dai L., Qi S., Li J., Colak G., Chen Y. (2016). Metabolic Regulation of Gene Expression by Histone Lysine β-Hydroxybutyrylation. Mol. Cell.

[48] Boison D. (2017). New insights into the mechanisms of the ketogenic diet. Curr. Opin. Neurol..

[49] Liu K., Li F., Sun Q., Lin N., Han H., You K., Tian F., Mao Z., Li T., Tong T. (2019). p53 β-hydroxybutyrylation attenuates p53 activity. Cell Death Dis..

[50] Zhang H., Tang K., Ma J., Zhou L., Liu J., Zeng L., Zhu L., Xu P., Chen J., Wei K. (2020). Ketogenesis-generated β-hydroxybutyrate is an epigenetic regulator of CD8. Nat. Cell Biol..

[51] Dąbek A., Wojtala M., Pirola L., Balcerczyk A. (2020). Modulation of Cellular Biochemistry, Epigenetics and Metabolomics by Ketone Bodies. Implications of the Ketogenic Diet in the Physiology of the Organism and Pathological States. Nutrients.

[52] Nishitani S., Fukuhara A., Shin J., Okuno Y., Otsuki M., Shimomura I. (2018). Metabolomic and microarray analyses of adipose tissue of dapagliflozin-treated mice, and effects of 3-hydroxybutyrate on induction of adiponectin in adipocytes. Sci. Rep..

[53] Ohashi K., Ouchi N., Matsuzawa Y. (2012). Anti-inflammatory and anti-atherogenic properties of adiponectin. Biochimie.

[54] Zhao S., Zhang X., Li H. (2018). Beyond histone acetylation-writing and erasing histone acylations. Curr. Opin. Struct. Biol..

[55] Chen X.F., Chen X., Tang X. (2020). Short-chain fatty acid, acylation and cardiovascular diseases. Clin. Sci..

[56] Zhang X., Cao R., Niu J., Yang S., Ma H., Zhao S., Li H. (2019). Molecular basis for hierarchical histone de-β-hydroxybutyrylation by SIRT3. Cell Discov..

[57] Hyun K., Jeon J., Park K., Kim J. (2017). Writing, erasing and reading histone lysine methylations. Exp. Mol. Med..

[58] Filippakopoulos P., Knapp S. (2014). Targeting bromodomains: Epigenetic readers of lysine acetylation. Nat. Rev. Drug Discov..

[59] Belkina A.C., Denis G.V. (2012). BET domain co-regulators in obesity, inflammation and cancer. Nat. Rev. Cancer.

[60] Wang Y.Z., Xu W.W., Zhu D.Y., Zhang N., Wang Y.L., Ding M., Xie X.M., Sun L.L., Wang X.X. (2018). Specific expression network analysis of diabetic nephropathy kidney tissue revealed key methylated sites. J. Cell Physiol..

[61] Ko Y.A., Mohtat D., Suzuki M., Park A.S.D., Izquierdo M.C., Han S.Y., Kang H.M., Si H., Hostetter T., Pullman J.M. (2013). Cytosine methylation changes in enhancer regions of core pro-fibrotic genes characterize kidney fibrosis development. Genome Biol..

[62] Wing M.R., Devaney J.M., Joffe M.M., Xie D., Feldman H.I., Dominic E.A., Guzman N.J., Ramezani A., Susztak K., Herman J.G. (2014). DNA methylation profile associated with rapid decline in kidney function: Findings from the CRIC study. Nephrol. Dial. Transplant..

[63] Marumo T., Yagi S., Kawarazaki W., Nishimoto M., Ayuzawa N., Watanabe A., Ueda K., Hirahashi J., Hishikawa K., Sakurai H. (2015). Diabetes Induces Aberrant DNA Methylation in the Proximal Tubules of the Kidney. J. Am. Soc. Nephrol..

[64] Sharma I., Dutta R.K., Singh N.K., Kanwar Y.S. (2017). High Glucose-Induced Hypomethylation Promotes Binding of Sp-1 to Myo-Inositol Oxygenase: Implication in the Pathobiology of Diabetic Tubulopathy. Am. J. Pathol.

[65] Li Z., Chen H., Zhong F., Zhang W., Lee K., He J.C. (2019). Expression of Glutamate Receptor Subtype 3 Is Epigenetically Regulated in Podocytes under Diabetic Conditions. Kidney Dis (Basel).

[66] Ling L., Chen L., Zhang C., Gui S., Zhao H., Li Z. (2018). 2018 High glucose induces podocyte epithelial-to-mesenchymal transition by demethylation-mediated enhancement of MMP9 expression. Mol. Med. Rep..

[67] Hayashi K., Sasamura H., Nakamura M., Azegami T., Oguchi H., Sakamaki Y., Itoh H. (2014). KLF4-dependent epigenetic remodeling modulates podocyte phenotypes and attenuates proteinuria. J. Clin. Investig..

[68] Hishikawa A., Hayashi K., Abe T., Kaneko M., Yokoi H., Azegami T., Nakamura M., Yoshimoto N., Kanda T., Sakamaki Y. (2019). Decreased KAT5 Expression Impairs DNA Repair and Induces Altered DNA Methylation in Kidney Podocytes. Cell Rep..

[69] Hasegawa K., Wakino S., Simic P., Sakamaki Y., Minakuchi H., Fujimura K., Hosoya K., Komatsu M., Kaneko Y., Kanda T. (2013). Renal tubular Sirt1 attenuates diabetic albuminuria by epigenetically suppressing Claudin-1 overexpression in podocytes. Nat. Med..

[70] Zhang H., Li A., Zhang W., Huang Z., Wang J., Yi B. (2016). High glucose-induced cytoplasmic translocation of Dnmt3a contributes to CTGF hypo-methylation in mesangial cells. Biosci. Rep..

[71] Li Y., Ren D., Shen Y., Zheng X., Xu G. (2020). Altered DNA methylation of TRIM13 in diabetic nephropathy suppresses mesangial collagen synthesis by promoting ubiquitination of CHOP. EBioMedicine.

[72] Gondaliya P., Dasare A., Srivastava A., Kalia K. (2019). Correction: miR29b regulates aberrant methylation in In-Vitro diabetic nephropathy model of renal proximal tubular cells. PLoS ONE.

[73] Richter K., Konzack A., Pihlajaniemi T., Heljasvaara R., Kietzmann T. (2015). Redox-fibrosis: Impact of TGFβ1 on ROS generators, mediators and functional consequences. Redox Biol..

[74] Oba S., Ayuzawa N., Nishimoto M., Kawarazaki W., Ueda K., Hirohama D., Kawakami-Mori F., Shimosawa T., Marumo T., Fujita T. (2018). Aberrant DNA methylation of Tgfb1 in diabetic kidney mesangial cells. Sci Rep..

[75] Yang L., Zhang Q., Wu Q., Yu J., Mu J., Zhang J., Zeng W., Feng B. (2018). Effect of TET2 on the pathogenesis of diabetic nephropathy through activation of transforming growth factor β1 expression via DNA demethylation. Life Sci..

[76] Yin S., Zhang Q., Yang J., Lin W., Li Y., Chen F., Cao W. (2017). TGFβ-incurred epigenetic aberrations of miRNA and DNA methyltransferase suppress Klotho and potentiate renal fibrosis. Biochim. Biophys. Acta.

[77] Zhang L., Zhang Q., Liu S., Chen Y., Li R., Lin T., Yu C., Zhang H., Huang Z., Zhao X. (2017). DNA methyltransferase 1 may be a therapy target for attenuating diabetic nephropathy and podocyte injury. Kidney Int..

[78] Chang Y.T., Yang C.C., Pan S.Y., Chou Y.H., Chang F.C., Lai C.F., Tsai M.H., Hsu H.L., Lin C.H., Chiang W.C. (2016). DNA methyltransferase inhibition restores erythropoietin production in fibrotic murine kidneys. J. Clin. Investig..

[79] Xiao X., Tang W., Yuan Q., Peng L., Yu P. (2015). Epigenetic repression of Krüppel-like factor 4 through Dnmt1 contributes to EMT in renal fibrosis. Int. J. Mol. Med..

[80] Li H., Zhang W., Zhong F., Das G.C., Xie Y., Li Z., Cai W., Jiang G., Choi J., Sidani M. (2018). Epigenetic regulation of RCAN1 expression in kidney disease and its role in podocyte injury. Kidney Int..

[81] Chen G., Chen H., Ren S., Xia M., Zhu J., Liu Y., Zhang L., Tang L., Sun L., Liu H. (2019). Aberrant DNA methylation of mTOR pathway genes promotes inflammatory activation of immune cells in diabetic kidney disease. Kidney Int..

[82] Tampe B., Tampe D., Müller C.A., Sugimoto H., LeBleu V., Xu X., Müller G.A., Zeisberg E.M., Kalluri R., Zeisberg M. (2014). Tet3-mediated hydroxymethylation of epigenetically silenced genes contributes to bone morphogenic protein 7-induced reversal of kidney fibrosis. J. Am. Soc. Nephrol..

[83] Lin Y.J., Zhen Y.Z., Wei J.B., Wei J., Dai J., Gao J.L., Li K.J., Hu G. (2017). Rhein lysinate protects renal function in diabetic nephropathy of KK/HlJ mice. Exp. Therapeutic Med..

[84] Zhang Q., Liu L., Lin W., Yin S., Duan A., Liu Z., Cao W. (2017). Rhein reverses Klotho repression via promoter demethylation and protects against kidney and bone injuries in mice with chronic kidney disease. Kidney Int..

[85] Sanchez-Niño M.D., Fernandez-Fernandez B., Ortiz A. (2020). Klotho, the elusive kidney-derived anti-ageing factor. Clin. Kidney J..

[86] Barrera-Chimal J., Jaisser F. (2020). Pathophysiologic mechanisms in diabetic kidney disease: A focus on current and future therapeutic targets. Diabetes Obes. Metab..

[87] Ochoa-Rosales C., Portilla-Fernandez E., Nano J., Wilson R., Lehne B., Mishra P.P., Gao X., Ghanbari M., Rueda-Ochoa O.L., Juvinao-Quintero D. (2020). Epigenetic Link Between Statin Therapy and Type 2 Diabetes. Diabetes Care.

[88] Gondaliya P., Dasare A., Srivastava A., Kalia K. (2018). miR29b regulates aberrant methylation in In-Vitro diabetic nephropathy model of renal proximal tubular cells. PLoS ONE.

[89] Kato M., Natarajan R. (2019). Epigenetics and epigenomics in diabetic kidney disease and metabolic memory. Nat. Rev. Nephrol..

[90] Yu C., Zhuang S. (2019). Histone Methyltransferases as Therapeutic Targets for Kidney Diseases. Front. Pharmacol..

[91] Miao F., Wu X., Zhang L., Yuan Y.C., Riggs A.D., Natarajan R. (2007). Genome-wide analysis of histone lysine methylation variations caused by diabetic conditions in human monocytes. J. Biol. Chem..

[92] Miao F., Smith D.D., Zhang L., Min A., Feng W., Natarajan R. (2008). Lymphocytes from patients with type 1 diabetes display a distinct profile of chromatin histone H3 lysine 9 dimethylation: An epigenetic study in diabetes. Diabetes.

[93] Miao F., Chen Z., Genuth S., Paterson A., Zhang L., Wu X., Li S.M., Cleary P., Riggs A., Harlan D.M. (2014). Evaluating the role of epigenetic histone modifications in the metabolic memory of type 1 diabetes. Diabetes.

[94] Ninichuk V., Kulkarni O., Clauss S., Anders H. (2007). Tubular atrophy, interstitial fibrosis, and inflammation in type 2 diabetic db/db mice. An accelerated model of advanced diabetic nephropathy. Eur. J. Med. Res..

[95] Sayyed S.G., Gaikwad A.B., Lichtnekert J., Kulkarni O., Eulberg D., Klussmann S., Tikoo K., Anders H.J. (2010). Progressive glomerulosclerosis in type 2 diabetes is associated with renal histone H3K9 and H3K23 acetylation, H3K4 dimethylation and phosphorylation at serine 10. Nephrol. Dial. Transplant..

[96] Ninichuk V., Clauss S., Kulkarni O., Schmid H., Segerer S., Radomska E., Eulberg D., Buchner K., Selve N., Klussmann S. (2008). Late onset of Ccl2 blockade with the Spiegelmer mNOX-E36-3′PEG prevents glomerulosclerosis and improves glomerular filtration rate in db/db mice. Am. J. Pathol..

[97] Komers R., Mar D., Denisenko O., Xu B., Oyama T.T., Bomsztyk K. (2013). Epigenetic changes in renal genes dysregulated in mouse and rat models of type 1 diabetes. Lab. Investig..

[98] Majumder S., Thieme K., Batchu S.N., Alghamdi T.A., Bowskill B.B., Kabir M.G., Liu Y., Advani S.L., White K.E., Geldenhuys L. (2018). Shifts in podocyte histone H3K27me3 regulate mouse and human glomerular disease. J. Clin. Investig..

[99] Jia Y., Reddy M.A., Das S., Oh H.J., Abdollahi M., Yuan H., Zhang E., Lanting L., Wang M., Natarajan R. (2019). Dysregulation of histone H3 lysine 27 trimethylation in transforming growth factor-β1-induced gene expression in mesangial cells and diabetic kidney. J. Biol. Chem..

[100] Siddiqi F.S., Majumder S., Thai K., Abdalla M., Hu P., Advani S.L., White K.E., Bowskill B.B., Guarna G., dos Santos C.C. (2016). The Histone Methyltransferase Enzyme Enhancer of Zeste Homolog 2 Protects against Podocyte Oxidative Stress and Renal Injury in Diabetes. J. Am. Soc. Nephrol..

[101] Lin S.H., Ho W.T., Wang Y.T., Chuang C.T., Chuang L.Y., Guh J.Y. (2016). Histone methyltransferase Suv39h1 attenuates high glucose-induced fibronectin and p21(WAF1) in mesangial cells. Int. J. Biochem. Cell Biol..

[102] Goru S.K., Kadakol A., Pandey A., Malek V., Sharma N., Gaikwad A.B. (2016). Histone H2AK119 and H2BK120 mono-ubiquitination modulate SET7/9 and SUV39H1 in type 1 diabetes-induced renal fibrosis. Biochem. J..

[103] Sun G., Reddy M.A., Yuan H., Lanting L., Kato M., Natarajan R. (2010). Epigenetic histone methylation modulates fibrotic gene expression. J. Am. Soc. Nephrol..

[104] Li Y., Reddy M.A., Miao F., Shanmugam N., Yee J.K., Hawkins D., Ren B., Natarajan R. (2008). Role of the histone H3 lysine 4 methyltransferase, SET7/9, in the regulation of NF-kappaB-dependent inflammatory genes. Relevance to diabetes and inflammation. J. Biol. Chem..

[105] Paneni F., Costantino S., Battista R., Castello L., Capretti G., Chiandotto S., Scavone G., Villano A., Pitocco D., Lanza G. (2015). Adverse epigenetic signatures by histone methyltransferase Set7 contribute to vascular dysfunction in patients with type 2 diabetes mellitus. Circ. Cardiovasc. Genet..

[106] Reddy M.A., Sumanth P., Lanting L., Yuan H., Wang M., Mar D., Alpers C.E., Bomsztyk K., Natarajan R. (2014). Losartan reverses permissive epigenetic changes in renal glomeruli of diabetic db/db mice. Kidney Int..

[107] Villeneuve L.M., Natarajan R. (2011). Epigenetic mechanisms. Contrib. Nephrol..

[108] Villeneuve L.M., Natarajan R. (2010). The role of epigenetics in the pathology of diabetic complications. Am. J. Physiol.-Ren. Physiol..

[109] Tonna S., El-Osta A., Cooper M.E., Tikellis C. (2010). Metabolic memory and diabetic nephropathy: Potential role for epigenetic mechanisms. Nat. Rev. Nephrol..

[110] Hills C.E., Squires P.E. (2010). TGF-beta1-induced epithelial-to-mesenchymal transition and therapeutic intervention in diabetic nephropathy. Am. J. Nephrol..

[111] Loeffler I., Wolf G. (2015). Epithelial-to-Mesenchymal Transition in Diabetic Nephropathy: Fact or Fiction?. Cells.

[112] Kolset S.O., Reinholt F.P., Jenssen T. (2012). Diabetic nephropathy and extracellular matrix. J. Histochem. Cytochem..

[113] Wang Y., Luo M., Wu H., Wu H., Kong L., Xin Y., Cui W., Zhao Y., Wang J., Liang G. (2015). Novel curcumin analog C66 prevents diabetic nephropathy via JNK pathway with the involvement of p300/CBP-mediated histone acetylation. Biochim. Biophys. Acta.

[114] Yuan H., Reddy M.A., Sun G., Lanting L., Wang M., Kato M., Natarajan R. (2013). Involvement of p300/CBP and epigenetic histone acetylation in TGF-β1-mediated gene transcription in mesangial cells. Am. J. Physiol.-Ren.Physiol..

[115] Das F., Ghosh-Choudhury N., Venkatesan B., Li X., Mahimainathan L., Choudhury G.G. (2008). Akt kinase targets association of CBP with SMAD 3 to regulate TGFbeta-induced expression of plasminogen activator inhibitor-1. J. Cell Physiol..

[116] Kato M., Dang V., Wang M., Park J.T., Deshpande S., Kadam S., Mardiros A., Zhan Y., Oettgen P., Putta S. (2013). TGF-β induces acetylation of chromatin and of Ets-1 to alleviate repression of miR-192 in diabetic nephropathy. Sci. Signal..

[117] Ghosh A.K., Bhattacharyya S., Lafyatis R., Farina G., Yu J., Thimmapaya B., Wei J., Varga J. (2013). p300 is elevated in systemic sclerosis and its expression is positively regulated by TGF-β: Epigenetic feed-forward amplification of fibrosis. J. Investig. Dermatol.

[118] Kanamaru Y., Nakao A., Tanaka Y. (2003). Involvement of p300 in TGF-beta/Smad-pathway-mediated alpha2(I) collagen expression in mouse mesangial cells. Nephron Exp. Nephrol..

[119] Malek V., Sharma N., Gaikwad A.B. (2019). Histone Acetylation Regulates Natriuretic Peptides and Neprilysin Gene Expressions in Diabetic Cardiomyopathy and Nephropathy. Curr. Mol. Pharmacol..

[120] Miao F., Gonzalo I.G., Lanting L., Natarajan R. (2004). In vivo chromatin remodeling events leading to inflammatory gene transcription under diabetic conditions. J. Biol. Chem..

[121] De Marinis Y., Cai M., Bompada P., Atac D., Kotova O., Johansson M.E., Garcia-Vaz E., Gomez M.F., Laakso M., Groop L. (2016). Epigenetic regulation of the thioredoxin-interacting protein (TXNIP) gene by hyperglycemia in kidney. Kidney Int..

[122] Chen H., Li J., Jiao L., Petersen R.B., Li J., Peng A., Zheng L., Huang K. (2014). Apelin inhibits the development of diabetic nephropathy by regulating histone acetylation in Akita mouse. J. Physiol..

[123] Hong Q., Zhang L., Das B., Li Z., Liu B., Cai G., Chen X., Chuang P.Y., He J.C., Lee K. (2018). Increased podocyte Sirtuin-1 function attenuates diabetic kidney injury. Kidney Int..

[124] Noh H., Oh E.Y., Seo J.Y., Yu M.R., Kim Y.O., Ha H., Lee H.B. (2009). Histone deacetylase-2 is a key regulator of diabetes- and transforming growth factor-beta1-induced renal injury. Am. J. Physiol.-Ren. Physiol..

[125] Sun X.Y., Qin H.J., Zhang Z., Xu Y., Yang X.C., Zhao D.M., Li X.N., Sun L.K. (2016). Valproate attenuates diabetic nephropathy through inhibition of endoplasmic reticulum stress-induced apoptosis. Mol. Med. Rep..

[126] Tikoo K., Meena R.L., Kabra D.G., Gaikwad A.B. (2008). Change in post-translational modifications of histone H3, heat-shock protein-27 and MAP kinase p38 expression by curcumin in streptozotocin-induced type I diabetic nephropathy. Br. J. Pharmacol..

[127] Yang Y., Liu K., Liang Y., Chen Y., Chen Y., Gong Y. (2015). Histone acetyltransferase inhibitor C646 reverses epithelial to mesenchymal transition of human peritoneal mesothelial cells via blocking TGF-β1/Smad3 signaling pathway in vitro. Int. J. Clin. Exp. Pathol..

[128] Sanz A.B., Ruiz-Andres O., Sanchez-Niño M.D., Ruiz-Ortega M., Ramos A.M., Ortiz A. (2016). Out of the TWEAKlight: Elucidating the Role of Fn14 and TWEAK in Acute Kidney Injury. Semin. Nephrol..

[129] Obokata M., Negishi K., Sunaga H., Ishida H., Ito K., Ogawa T., Iso T., Ando Y., Kurabayashi M. (2017). Association Between Circulating Ketone Bodies and Worse Outcomes in Hemodialysis Patients. J. Am. Heart Assoc..

[130] Poplawski M.M., Mastaitis J.W., Isoda F., Grosjean F., Zheng F., Mobbs C.V. (2011). Reversal of diabetic nephropathy by a ketogenic diet. PLoS ONE.

[131] Khan S., Jena G. (2014). Sodium butyrate, a HDAC inhibitor ameliorates eNOS, iNOS and TGF-β1-induced fibrogenesis, apoptosis and DNA damage in the kidney of juvenile diabetic rats. Food Chem. Toxicol..

[132] Locatelli M., Zoja C., Zanchi C., Corna D., Villa S., Bolognini S., Novelli R., Perico L., Remuzzi G., Benigni A. (2020). Manipulating Sirtuin 3 pathway ameliorates renal damage in experimental diabetes. Sci. Rep..

[133] Zhang L., Liu J., Zhou F., Wang W., Chen N. (2018). PGC-1α ameliorates kidney fibrosis in mice with diabetic kidney disease through an antioxidative mechanism. Mol. Med. Rep..

[134] Chow F.Y., Nikolic-Paterson D.J., Ozols E., Atkins R.C., Rollin B.J., Tesch G.H. (2006). Monocyte chemoattractant protein-1 promotes the development of diabetic renal injury in streptozotocin-treated mice. Kidney Int..

[135] Fontecha-Barriuso M., Martin-Sanchez D., Martinez-Moreno J.M., Monsalve M., Ramos A.M., Sanchez-Niño M.D., Ruiz-Ortega M., Ortiz A., Sanz A.B. (2020). The Role of PGC-1α and Mitochondrial Biogenesis in Kidney Diseases. Biomolecules.

[136] Morgado-Pascual J.L., Rayego-Mateos S., Tejedor L., Suarez-Alvarez B., Ruiz-ortega M. (2019). Bromodomain and Extraterminal Proteins as Novel Epigenetic Targets for Renal Diseases. Front. Pharmacol..

[137] Morgado-Pascual J.L., Marchant V., Rodrigues-Diez R., Dolade N., Suarez-Alvarez B., Kerr B., Valdivielso J.M., Ruiz-Ortega M., Rayego-Mateos S. (2018). Epigenetic Modification Mechanisms Involved in Inflammation and Fibrosis in Renal Pathology. Med. Inflamm..

[138] Lovén J., Hoke H.A., Lin C.Y., Lau A., Orlando D.A., Vakoc C.R., Bradner J.E., Lee T.I., Young R.A. (2013). Selective inhibition of tumor oncogenes by disruption of super-enhancers. Cell.

[139] Suarez-Alvarez B., Rodriguez R.M., Ruiz-Ortega M., Lopez-Larrea C. (2017). BET Proteins: An Approach to Future Therapies in Transplantation. Am. J. Transplant..

[140] Amir-Zilberstein L., Ainbinder E., Toube L., Yamaguchi Y., Handa H., Dikstein R. (2007). regulation of NF-kappaB by elongation factors is determined by core promoter type. Mol. Cell. Biol..

[141] Huang B., Yang X.D., Zhou M.M., Ozato K., Chen L.F. (2009). Brd4 coactivates transcriptional activation of NF-kappaB via specific binding to acetylated RelA. Mol. Cell. Biol..

[142] Zou Z., Huang B., Wu X., Zhang H., Qi J., Bradner J., Nair S., Chen L.F. (2014). Brd4 maintains constitutively active NF-κB in cancer cells by binding to acetylated RelA. Oncogene.

[143] Suarez-Alvarez B., Morgado-Pascual J.L., Rayego-Mateos S., Rodriguez R.M., Rodrigues-Diez R., Cannata-Ortiz P., Sanz A.B., Egido J., Tharaux P.L., Ortiz A. (2017). Inhibition of Bromodomain and Extraterminal Domain Family Proteins Ameliorates Experimental Renal Damage. J. Am. Soc. Nephrol..

[144] Sanchez-Niño M.D., Benito-Martin A., Ortiz A. (2010). New paradigms in cell death in human diabetic nephropathy. Kidney Int..

[145] Lavoz C., Rayego-Mateos S., Orejudo M., Opazo-Ríos L., Marchant V., Marquez-Exposito L., Tejera-Muñoz A., Navarro-González J.F., Droguett A., Ortiz A. (2020). Could IL-17A Be a Novel Therapeutic Target in Diabetic Nephropathy?. J. Clin. Med..

[146] Thompson P.J., Shah A., Apostolopolou H., Bhushan A. (2019). BET Proteins Are Required for Transcriptional Activation of the Senescent Islet Cell Secretome in Type 1 Diabetes. Int. J. Mol. Sci..

[147] Deeney J.T., Belkina A.C., Shirihai O.S., Corkey B.E., Denis G.V. (2016). BET Bromodomain Proteins Brd2, Brd3 and Brd4 Selectively Regulate Metabolic Pathways in the Pancreatic β-Cell. PLoS ONE.

[148] Huijbregts L., Petersen M.B.K., Berthault C., Hansson M., Aiello V., Rachdi L., Grapin-Botton A., Honore C., Scharfmann R. (2019). Bromodomain and Extra Terminal Protein Inhibitors Promote Pancreatic Endocrine Cell Fate. Diabetes.

[149] Wang J., Hu J., Chen X., Huang C., Lin J., Shao Z., Gu M., Wu Y., Tian N., Gao W. (2019). BRD4 inhibition regulates MAPK, NF-κB signals, and autophagy to suppress MMP-13 expression in diabetic intervertebral disc degeneration. FASEB J..

[150] Guo M., Wang H.X., Chen W.J. (2018). BET-inhibition by JQ1 alleviates streptozotocin-induced diabetic cardiomyopathy. Toxicol. Appl. Pharmacol..

[151] Wang Q., Sun Y., Li T., Liu L., Zhao Y., Li L., Zhang L., Meng Y. (2019). Function of BRD4 in the pathogenesis of high glucose-induced cardiac hypertrophy. Mol. Med. Rep..

[152] Zuo H., Wang S., Feng J., Liu X. (2019). BRD4 contributes to high-glucose-induced podocyte injury by modulating Keap1/Nrf2/ARE signaling. Biochimie.

[153] Kulikowski E., Halliday C., Johansson J., Sweeney M., Lebioda K., Wong N., Haarhaus M., Brandenburg V., Beddhu S., Tonelli M. (2018). Apabetalone Mediated Epigenetic Modulation is Associated with Favorable Kidney Function and Alkaline Phosphatase Profile in Patients with Chronic Kidney Disease. Kidney Blood Press. Res..

[154] Sumida K., Molnar M.Z., Potukuchi P.K., Thomas F., Lu J.L., Obi Y., Rhee C.M., Streja E., Yamagata K., Kalantar-Zadeh K. (2018). Prognostic significance of pre-end-stage renal disease serum alkaline phosphatase for post-end-stage renal disease mortality in late-stage chronic kidney disease patients transitioning to dialysis. Nephrol. Dial. Transplant..

[155] Regidor D.L., Kovesdy C.P., Mehrotra R., Rambod M., Jing J., McAllister C.J., Van Wyck D., Kopple J.D., Kalantar-Zadeh K. (2008). Serum alkaline phosphatase predicts mortality among maintenance hemodialysis patients. J. Am. Soc. Nephrol..

[156] Ray K.K., Nicholls S.J., Ginsberg H.D., Johansson J.O., Kalantar-Zadeh K., Kulikowski E., Toth P.P., Wong N., Cummings J.L., Sweeney M. (2019). Effect of selective BET protein inhibitor apabetalone on cardiovascular outcomes in patients with acute coronary syndrome and diabetes: Rationale, design, and baseline characteristics of the BETonMACE trial. Am. Heart J..

[157] Ray K.K., Nicholls S.J., Buhr K.A., Ginsberg H.N., Johansson J.O., Kalantar-Zadeh K., Kulikowski E., Toth P.P., Wong N., Sweeney M. (2020). Effect of Apabetalone Added to Standard Therapy on Major Adverse Cardiovascular Events in Patients With Recent Acute Coronary Syndrome and Type 2 Diabetes: A Randomized Clinical Trial. JAMA.

[158] Sanchez-Niño M.D., Carpio D., Sanz A.B., Ruiz-Ortega M., Mezzano S., Ortiz A. (2015). Lyso-Gb3 activates Notch1 in human podocytes. Hum. Mol. Genet..

[159] Sanchez-Niño M.D., Sanz A.B., Carrasco S., Saleem M.A., Mathieson P.W., Valdivielso J.M., Ruiz-Ortega M., Egido J., Ortiz A. (2011). Globotriaosylsphingosine actions on human glomerular podocytes: Implications for Fabry nephropathy. Nephrol. Dial. Transplant..

[160] Aguilera-Correa J.J., Madrazo-Clemente P., Martínez-Cuesta M.D.C., Peláez C., Ortiz A., Sánchez-Niño M.D., Esteban J., Requena T. (2019). Lyso-Gb3 modulates the gut microbiota and decreases butyrate production. Sci. Rep...

[161] van Bommel E.J.M., Lytvyn Y., Perkins B.A., Soleymanlou N., Fagan N.M., Koitka-Weber A., Joles J.A., Cherney D.Z., van Raalte D.H. (2020). Renal hemodynamic effects of sodium-glucose cotransporter 2 inhibitors in hyperfiltering people with type 1 diabetes and people with type 2 diabetes and normal kidney function. Kidney Int..

[162] Soler M.J., Porrini E., Fernandez-Fernandez B., Ortiz A. (2020). SGLT2i and postglomerular vasodilation. Kidney Int..

[163] Ferrannini E., Mark M., Mayoux E. (2016). CV Protection in the EMPA-REG OUTCOME Trial: A “Thrifty Substrate” Hypothesis. Diabetes Care.

[164] Solini A., Seghieri M., Giannini L., Biancalana E., Parolini F., Rossi C., Dardano A., Taddei S., Ghiadoni L., Bruno R.M. (2019). The Effects of Dapagliflozin on Systemic and Renal Vascular Function Display an Epigenetic Signature. J. Clin. Endocrinol. Metab..

[165] Fontecha-Barriuso M., Martín-Sánchez D., Martinez-Moreno J.M., Carrasco S., Ruiz-Andrés O., Monsalve M., Sanchez-Ramos C., Gómez M.J., Ruiz-Ortega M., Sánchez-Niño M.D. (2019). PGC-1α deficiency causes spontaneous kidney inflammation and increases the severity of nephrotoxic AKI. J. Pathol..

[166] Ruiz-Andres O., Suarez-Alvarez B., Sánchez-Ramos C., Monsalve M., Sanchez-Niño M.D., Ruiz-Ortega M., Egido J., Ortiz A., Sanz A.B. (2015). The inflammatory cytokine TWEAK decreases PGC-1α expression and mitochondrial function in acute kidney injury. Kidney Int..

[167] Tran M., Tam D., Bardia A., Bhasin M., Rowe G.C., Kher A., Zsengeller Z.K., Akhavan-Sharif M.R., Khankin E.V., Saintgeniez M. (2011). PGC-1α promotes recovery after acute kidney injury during systemic inflammation in mice. J. Clin. Investig..

